# Data-Independent Acquisition-Based Proteome and Phosphoproteome Profiling Reveals Early Protein Phosphorylation and Dephosphorylation Events in *Arabidopsis* Seedlings upon Cold Exposure

**DOI:** 10.3390/ijms222312856

**Published:** 2021-11-27

**Authors:** Jinjuan Tan, Zhongjing Zhou, Hanqian Feng, Jiayun Xing, Yujie Niu, Zhiping Deng

**Affiliations:** 1 State Key Laboratory for Managing Biotic and Chemical Threats to the Quality and Safety of Agro-Products, Institute of Virology and Biotechnology, Zhejiang Academy of Agricultural Sciences, Hangzhou 310021, China; jinjuantan@zaas.ac.cn (J.T.); zhouzj@zaas.ac.cn (Z.Z.); fenghq@zaas.ac.cn (H.F.); niuyj1990@163.com (Y.N.); 2 College of Agronomy, Hunan Agricultural University, Changsha 410128, China; jyxing11@126.com

**Keywords:** data-independent acquisition (DIA), proteome, phosphoproteome, cold stress, parallel reaction monitoring (PRM), MAP kinase, transcription factor, *Arabidopsis*

## Abstract

Protein phosphorylation plays an important role in mediating signal transduction in cold response in plants. To better understand how plants sense and respond to the early temperature drop, we performed data-independent acquisition (DIA) method-based mass spectrometry analysis to profile the proteome and phosphoproteome of *Arabidopsis* seedlings upon cold stress in a time-course manner (10, 30 and 120 min of cold treatments). Our results showed the rapid and extensive changes at the phosphopeptide levels, but not at the protein abundance levels, indicating cold-mediated protein phosphorylation and dephosphorylation events. Alteration of over 1200 proteins at phosphopeptide levels were observed within 2 h of cold treatment, including over 140 kinases, over 40 transcriptional factors and over 40 E3 ligases, revealing the complexity of regulation of cold adaption. We summarized cold responsive phosphoproteins involved in phospholipid signaling, cytoskeleton reorganization, calcium signaling, and MAPK cascades. Cold-altered levels of 73 phosphopeptides (mostly novel cold-responsive) representing 62 proteins were validated by parallel reaction monitoring (PRM). In summary, this study furthers our understanding of the molecular mechanisms of cold adaption in plants and strongly supports that DIA coupled with PRM are valuable tools in uncovering early signaling events in plants.

## 1. Introduction

Cold stress, which is generally categorized into chilling stress (0–15 °C) and freezing stress (<0 °C), controls the geographical distribution, quality, and yield of crop plants. Plants from temperate regions, such as *Arabidopsis thaliana*, commonly have a certain level of basal freezing tolerance, which could be further enhanced by prior exposure to non-freezing low temperatures, a process known as cold acclimation [1]. Great progress has been made in elucidating the molecular mechanism of cold tolerance in plants [2,3,4]. The currently accepted hypothesis of cold signal perception and transduction in plants begins with a decrease in cell membrane fluidity and reorganization of the cytoskeleton, and then transient Ca^2+^ influx into cytoplasm occurs, and downstream responses are activated [3]. The C-repeat binding factors/drought response element binding factor 1s (CBFs/DREB1s)-dependent regulation is currently the best understood cold response pathway, and it is considered as a critical regulatory pathway in cold acclimation [5]. CBFs control the transcription regulation of a set of *cold-regulated* (*COR*) genes upon cold stress, which are known as CBF regulons. Many transcription factors contribute to the transcriptional regulation of *CBFs*, and Inducer of CBF expression 1 (ICE1) is currently the best characterized one [2,3,6].

Reversible protein phosphorylation is one of the most important post-translational modifications in eukaryotes which controls protein activity, subcellular localization, and interaction with other proteins. It plays an important role in the early cold adaption and the later attenuation of cold stimulus. For instance, Open stomata 1 (OST1)/SNF1-related protein kinase 2.6 (SnRK2.6) phosphorylates and stabilizes ICE1 and positively regulate cold signaling [7], while Mitogen activated protein kinase 3/6 (MPK3/6) and Brassinosteroid insensitive 2 (BIN2) negatively regulate cold tolerance by phosphorylating ICE1 and promoting its degradation [8,9,10]. Despite advances in understanding the molecular mechanisms of cold response in plants [2,3,4], our ability to improve cold tolerance in crops is limited and hampered by the complexity of cold tolerance in plants. The cold-mediated transcriptional network has been extensively studied [11,12], but the comprehensive analysis of cold-mediated protein post-translational regulation has only gained attention in recent years. Recently, a study focused on phosphorylation events of membrane proteins under short-time cold stress in *Arabidopsis* [13] and another study providing deep phosphoproteomic data concerning long time cold stress of tomato leaves [14] have greatly enlarged our understanding of cold response; however, systematically analysis of the early events induced by cold at deep-scale whole phosphoproteomic level in a time-course manner is still lacking.

In the past decade, great technological advances in liquid chromatography coupled with tandem mass spectrometry (LC-MS/MS) technology and bioinformatic tools have enabled comprehensive proteome and phosphoproteome profiling in eukaryotes. Data-independent acquisition (DIA)-based MS analysis has gained popularity in recent years due to its unbiased and comprehensive sampling of precursor ions for fragmentation compared to the traditional data-dependent acquisition (DDA) method with biased sampling [15,16,17], and thus has the advantage of more producible and accurate quantification with fewer missing values across samples and higher identification breadth. A comprehensive spectral library is usually required for DIA analysis, from which the single-MS spectra are matched to the library peptides, although it was shown that direct DIA without the library is feasible for global protein phosphorylation analysis [18]. DIA-based proteome profiling has been used widely in studying the biotic and abiotic stresses in plants [19,20,21], although DIA-based phosphoproteome profiling is lacking in plants.

Despite great progress in elucidating the molecular mechanism of cold tolerance in plants, our understanding of the specific cold response is still limited, especially in the early cold signal perception and transduction events. It is known that signal sensing and relay commonly happen rapidly, especially for the physical temperature signal that reaches the surface and interior of a cell almost simultaneously [22,23,24]. To determine the global alteration of proteins and protein phosphorylation levels upon cold treatment within minutes and hours, we performed DIA-MS based large-scale time-course proteomic and phosphoproteomic analyses to monitor protein/phosphopeptide abundance in *Arabidopsis* seedlings upon cold stimuli. Our results indicate that more than 400 phosphopeptides were significantly changed upon cold treatment as short as 10 min. On the contrary, only a few proteins (total protein) showed abundance alteration after 2 hours of cold treatment. Therefore, most of the changes at the phosphopeptide levels are likely at the protein phosphorylation levels, but not at the protein abundance levels. Based on the current model for cold signal sensing and transduction, we discussed the cold-responsive phosphoproteins involved in various signal transduction processes, including phospholipid signaling, cytoskeleton reorganization, Ca^2+^ sensoring, MAPK cascade signaling and downstream transcription regulation. Moreover, we validated over 70 cold-responsive phosphopeptides by parallel reaction monitoring (PRM), many of which include novel cold-responsive phosphosites (p-sites).

## 2. Results

### 2.1. Proteome and Phosphoproteome Analyses of Arabidopsis Seedlings upon Short-Time Cold Stress

**Figure 1:**
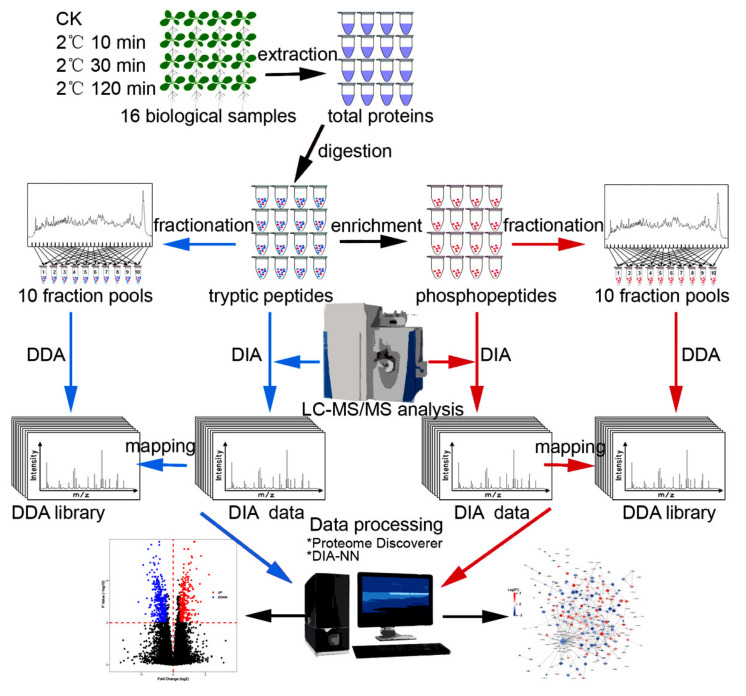
Schematic representation of the experimental workflow.

Spectral library-based DIA analyses were performed to profile proteome and phosphoproteome of *Arabidopsis* seedlings in response to cold treatment for 0, 10, 30, and 120 min, respectively (CK, CS10, CS30, and CS120), and each time point contains four biological replicates. A simple workflow of the proteomic and phosphoproteomic analyses are shown in Figure 1. The quantitative values of (phospho)peptides abundance was derived by peak intensity, and the abundance values of protein were calculated by summing up the most intense one to three peptides per protein. Since some of the peptides are shared among multiple proteins or protein isoforms, the quantified proteins mentioned in this study actually refer to protein groups, as in other proteomic literatures [25].

**Figure 2:**
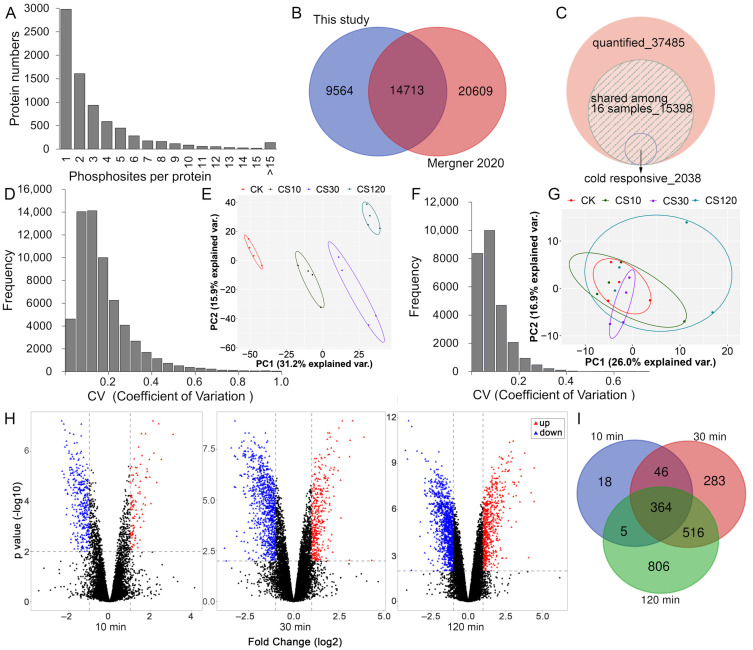
Quantitative time-course study of the proteome and phosphoproteome of *Arabidopsis* seedlings upon cold stress. (**A**) Numbers of proteins containing one or more p-sites. (**B**) Venn diagram comparing high-confidence p-sites in this study with class I p-sites identified in a recent Arabidopsis phosphoproteome [30]. (**C**) Number of total quantified and cold-responsive phosphopeptides. The cold-responsive phosphopeptides were determined using the cutoff of fold change > 2 or <0.5 and adjusted *p* value < 0.01. (**D**) Coefficient of variation (CV) values distribution calculated for each phosphopeptide for each four replicates. (**E**) PCA analysis of the phosphoproteomics data. (**F**) CV values distribution calculated for each protein for each four replicates. (**G**) PCA analysis of the global proteomics data. (**H**) Volcano plots showing upregulated and downregulated phosphopeptides in response to different periods of cold stress. (**I**) Venn diagram showing phosphopeptides significantly differentially regulated by different periods of cold stress.

Ga^3+^-based immobilized metal affinity chromatography (Ga-IMAC) was used to enrich phosphopeptides in this study since it was shown to be reproducible and specific in purifying phosphoproteins in *Arabidopsis* seedlings [26] and efficient in enriching phosphopeptides in *Arabidopsis* inflorescence tissues [27] and in rat pancreas [28]. A spectral library for phosphoproteome was constructed using Ga-IMAC enrichment followed by high-pH reversed-phase fractionation, LC-MS/MS analysis and identification by Proteome Discoverer (Figure 1 and Table S1, FDR < 1% for PSMs and peptide groups). We identified 47,362 phosphopeptides (or peptide isoforms), corresponding to 35,002 phosphopeptide groups, and most of the phosphopeptides were mono phosphorylated (85.9%), 12.1% were doubly phosphorylated, while 1.9% were triply phosphorylated. Among the 24,277 unique high-confidence p-sites (ptmRS site probability over 75%) [29], 86.9% sites of phosphorylation were localized at serine residues, 12.8% were at threonine residues, and 0.2% were at tyrosine residues, which showed a similar residues phosphorylation proportion to the recent report of *Arabidopsis* phosphoproteome (86.0% at serine, 13.64% at threonine and 0.36% at tyrosine residues) [30]. Approximately two-thirds of the identified phosphoproteins contained more than one p-sites (Figure 2A). Comparison of the p-sites identified in this study with a recent large-scale phosphoproteomics profiling in *Arabidopsis* [30] showed that only 61% of the high-confidence p-sites identified in this study were in the class I p-sites (localization probability over 0.75) [31], identified by Mergner et al. (Figure 2B and Table S2), supporting that Ga-IMAC and Fe-IMAC are complementary in providing a more comprehensive mapping of the phosphoproteome [32].

DIA-NN, an open-source deep-neural network-based software for DIA-based profiling of both proteome [33] and post-translationally-modified proteome [34], was used to analyze the DIA data in this study. With a global false discovery rate (FDR) < 1%, we totally quantified 37,485 phosphopeptides (28,501 p-sites), representing 7076 protein groups, and 15,398 phosphopeptides were identified in all 16 samples (Figure 2C and Table S3). Setting the coefficient of variation (CV) threshold at 20%, the cumulative fraction of CV values for replicates was 69.4% (Figure 2D), and the median CV values for replicates of CK, CS10, CS30, and CS120 were 13.8%, 15.9%, 16.6%, and 11.1% respectively (Figure S1A).

For the global proteome analysis, we identified 11,936 protein groups and 119,349 peptide groups in *Arabidopsis* seedlings by analyzing the DDA files from the fractionated peptides using Proteome Discoverer (Table S4, FDR < 1% for PSMs, peptide groups and protein levels) and built the spectral library with Spectronaut 13. Using DIA-NN to analyze the DIA data, we totally identified 8423 protein groups (FDR < 1%), and 6733 of them were shared among all 16 samples. The cumulative fraction of CV values above 20% was 93.3% (Figure 2F), and the median CV values for replicates of CK, CS10, CS30, and CS120 were 6.1%, 6.6%, 6.1%, and 10.5%, respectively (Figure S1B). These results indicated good reproducibility of the DIA-MS based proteomic and phosphoproteomic analyses. Principal component analysis (PCA, Figure 2E) of the phosphoproteomic data showed that the first component (PC1, 31.2%) clearly and sequentially separates the four sample groups, however, the PCA analysis of the proteomic data showed that the sample groups cannot be separated clearly (Figure 2G). These results suggested that cold treatment within 120 min significantly altered phosphoproteome, but not proteome, of the liquid-cultured *Arabidopsis* seedlings.

### 2.2. Identification of Early-Cold-Responsive Proteins

Total peptides/phosphopeptides quantified in all 16 samples were used for statistical analysis. Using cut-off criteria of adjusted *p* value < 0.01 and fold change >2 or <0.5, we identified 2038 early cold-responsive phosphopeptides (corresponding to 1208 protein groups) in at least one time-point of cold treatment, including 707 up-accumulated and 1334 down-accumulated phosphopeptides (Figure 2C,H and Table S5). The number of cold-responsive phosphopeptides increased along with the treatment time, and abundance levels of 433 phosphopeptides were significantly altered upon 10 min of cold stress (Figure 2H,I), suggesting a rapid cold response at protein phosphorylation level. Three phosphopepetides ascribed to exocyst complex component SEC15B, Nuclear DBF2-related 6 (NDR6) and TOM1-like protein 3 (TOL3) were included in both up- and down-accumulated phosphopeptide lists, and they all showed an initially downregulated and subsequently upregulated responsive profile (Figure S2 and Table S5). In addition, some phosphopeptides were also discussed in this study for their potential roles during cold adaption, even though their cold response were less strong (fold change >1.5 or <0.67 and adjusted *p* value < 0.05) (Table S6). Furthermore, phosphopeptides with missing values in all four biological replicates of control or cold treatment sample groups were included in Table S7 and selected ones were analyzed by PRM, as phosphopeptide signal of many important signaling proteins may not be detected due to low abundance.

Statistical analysis of the proteome dataset indicated that seven proteins showed significant abundance change within 120 min of cold treatment (adjusted *p* value < 0.01 and fold change >1.5 or <0.67) (Table 1), suggesting that the early cold response for proteins occurs mainly on post-translational modification or subcellular relocation, but not on total protein abundance level. We found that the Photosystem II Reaction Center Protein H (PSBH) and the major plastocyanin of PETE2 were the earliest detected cold-responsive proteins, and both of them showed significant abundance decrease at 30 min of cold treatment (Table 1), suggesting that photosynthesis inhibition may be one of the earliest biological processes in cold response.

### 2.3. Protein Phosphorylation and Dephosphorylation Response Showed Distinct Patterns in Biological Processes or Pathways

We found that dephosphorylation (down-accumulation) events occur more frequently than phosphorylation (up-accumulation) upon cold stress within 120 min, especially at the early cold response stage (76% of dephosphorylation events at 10 min) (Figure 2H). As the cold treatment period increased, the percentage of the down-accumulated phosphopeptides decreased (Figure 2H). Gene ontology (GO) enrichment analysis revealed that, even though proteins with phosphorylation or dephosphorylation events were both enriched in biological processes associated with protein phosphorylation and response to abscisic acid, those with phosphorylation events were uniquely enriched for the categories involved in signal transduction, RNA/mRNA splicing, respond to salt/osmotic stress, and protein translation/transport, whereas those with dephosphorylation events were uniquely enriched for the categories related to root hair elongation, cold acclimation, cellulose biosynthesis, and cell death (Figure S3A). Similarly, kyoto encyclopedia of genes and genomes (KEGG) pathway analysis indicated that proteins with phosphorylation or dephosphorylation events were both enriched in spliceosome and endocytosis and that the phosphorylation events were uniquely enriched for proteins in MAPK signaling and RNA transport, while the dephosphorylation events were significantly enriched in proteins involved in mRNA surveillance pathway (Figure S3D). These results suggested that, upon cold stimuli, protein phosphorylation response happened more frequently in RNA processing and signal transduction, while dephosphorylation response occurred more frequently in growth-associated processes. In addition, approximately 15% (180) of the cold-responsive phosphoproteins contain both phosphorylation and dephosphorylation events, the percentage of these proteins was increased along with the increasing cold treatment period (Figure S4A). GO and KEGG analyses of these proteins are shown in Figure S4B,C.

**Figure 3:**
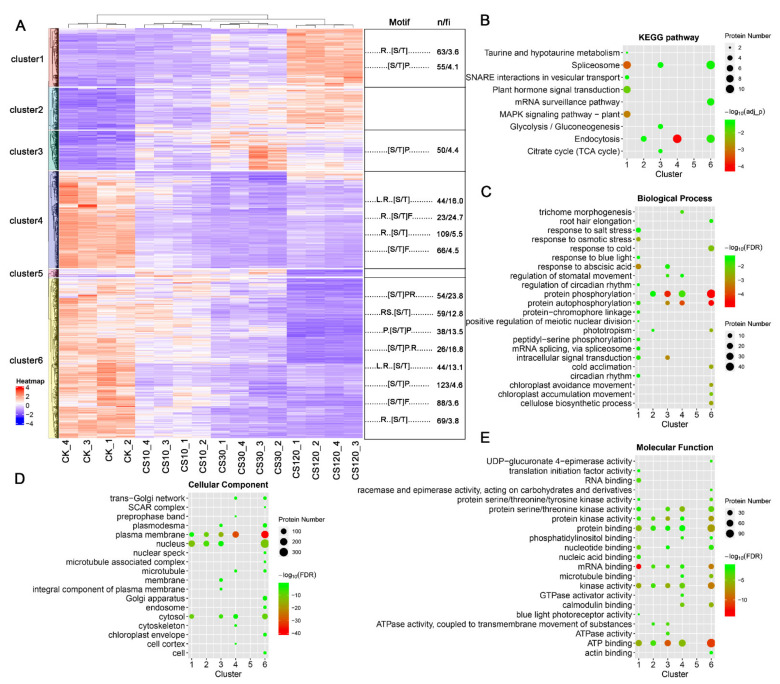
Clustering analysis of cold-responsive phosphopeptides. (**A**) HCA analysis of cold-responsive phosphopeptides (left) and phosphorylation site motif analysis of each cluster (right), “n” indicates the number of p-sites with the motif and “fi” is an indicator of the enrichment level of the extracted motifs and is calculated as (foreground matches/foreground size)/(background matches/background size). KEGG pathway enrichment (**B**) and GO enrichment analysis (**C**–**E**) of proteins with cold-responsive phosphopeptides belonging to each cluster were shown.

To determine the patterns of (de)phosphorylation response upon different periods of cold stress, the significantly responded phosphopeptides were grouped into six clusters using hierarchical clustering analysis (HCA) method (Figure 3A). Cluster 1, 2 or 3 include phosphopeptides upregulated during cold stress, while cluster 4, 5 and 6 include phosphopeptides downregulated by cold. Further analysis found that proteins of different clusters were enriched in different biological processes or pathways. For instance, proteins of cluster 1, which showed a steady increase of phosphopeptide levels along with cold stress time, were enriched in biological processes related to environmental signal/stress response and mRNA splicing, while proteins of cluster 6, which showed a contrary tendency of cluster 1, were significantly enriched in categories of protein phosphorylation/autophosphorylation and other events that differed from cluster 1 (Figure 3C). Only proteins of cluster 6 were significantly enriched in cold response/acclimation (Figure 3C). For pathway analysis, cluster 1 proteins were enriched in spliceosome, MAPK cascade signaling and hormone signaling, while cluster 4 proteins were mainly enriched in endocytosis (Figure 3B). These results suggested that cold-induced dynamic phosphorylation modification behave distinctly in different biological processes or pathways.

### 2.4. Motif Analysis of Sequences Flanking p-Sites of Cold-Responsive Phosphopeptides

Conserved motifs surrounding the phosphorylated amino acids provide important information concerning kinase-substrate specificities. Using an online tool MotifeR [35], we identified 191 phosphorylation motifs (169 motifs with score > 300) from our phosphoproteomic data (Table S8). Setting a cut-off threshold for motif score of 300, 12 phosphorylation motifs were significantly enriched from the 2038 cold-responsive phosphopeptides (Table S8). Besides, three phosphorylation motifs were enriched from the up-accumulated phosphopeptides in response to cold, while 11 motifs were enriched from the down-accumulated ones, indicating distinct modification profiles between the cold-induced phosphorylation and dephosphorylation events (Figure S5 and Table S8). Similarly, different phosphorylation motif categories were identified from cold-responsive phosphopeptides belonging to different HCA clusters (Figure 3A and Table S8). These results suggest that kinase/phosphatase activity perturbation is one of the key events triggered by cold stimuli. For instance, the kinases targeting motif ..[S/T]P... supports the active action of MAPKs, cyclin-dependent protein kinases (CDKs) and shaggy-like kinases (SLKs), and kinases targeting motif ..R..[S/T]... supports the action of SnRK2s, Ca^2+^-dependent protein kinases (CPKs), Ca^2+^/calmodulin dependent protein kinases (CaMKs) and CIPKs [36,37,38] in the early cold signal transduction.

### 2.5. Cold Stimuli Induced Dynamic Protein Phosphorylation Modification

**Figure 4:**
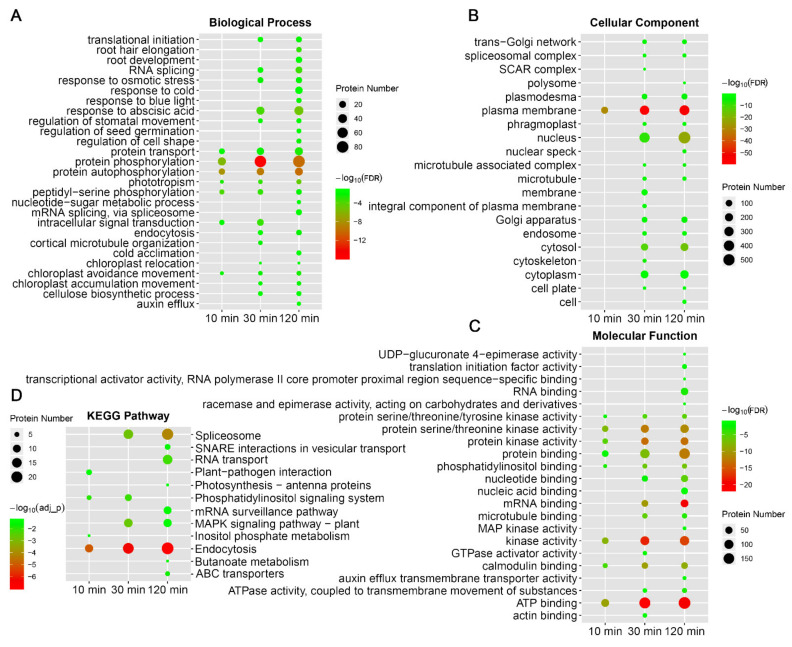
GO enrichment (**A**–**C**) and KEGG pathway (**D**) analysis of proteins with phosphopeptides significantly responsive to different time points of cold stress.

GO enrichment analyses suggested that proteins with cold-responsive phosphopeptides were initially (10-minute cold stress) enriched in biological processes involved in protein phosphorylation/autophosphorylation, intracellular signal transduction, protein transport, phototropism, and chloroplast movement, and then extended to abscisic acid/osmotic stress response, stomatal movement, cellulose biosynthesis, translation initiation, RNA splicing and endocytosis upon 30 min cold stress. When the cold treatment period increased to 120 min, more biological processes such as those involved in growth/development, bule light response, mRNA splicing, cold acclimation/response and nucleotide-sugar metabolism were enriched (Figure 4A). Similarly, as cold treatment time increased, the proteins with cold-responsive phosphopeptides were enriched in more GO items of cellular component or molecular function; for instance, proteins with RNA binding function and MAP kinase activity were enriched until 120 min of cold treatment (Figure 4B,C). KEGG pathway analysis indicated that the early cold responses involve pathways such as endocytosis and phosphatidylinositol signaling, and then more pathways were activated, including those related to RNA/protein processing and transport, MAPK signaling and photosynthesis (Figure 4D). These results indicated that cold-induced biological processes/pathways are sequentially activated along with the cold stress time. Overall, in the earlier stage, *Arabidopsis* seedlings sense and respond to cold by rapid signal transduction and activating energy/metabolite reallocation between growth and stress responses, suggested by the activation of biological processes/pathways involving in protein phosphorylation/trafficking and chloroplast movement. Then, in the later stage, more specific cold responses occur, such as stress/phytohormone response, protein/RNA processing, and growth/development regulation, and thus *Arabidopsis* seedlings convert survival strategies from growth to cold adaption.

### 2.6. Cold-Induced Phosphorylation and Dephosphorylation in Phospholipid Signaling Proteins

The lipid-derived signaling molecules, such as phosphoinositides (PIs) and phosphatidic acid (PA), account for less than 1% of the total biomembrane lipids but act as key metabolic intermediates and messengers in many biological processes [39,40,41]. The plant PIs are formed and degraded with a high turnover rate, and easily perturbed by environmental stimulation, and therefore enable rapid signal events upon stresses. Besides, the regulation of PIs under stresses might mainly occur on protein post-translational modification, such as phosphorylation, rather than on transcription level [42]. Here, in our results, the phosphatidylinositol (PtdIns) signaling system and inositol phosphate metabolism are enriched pathways among proteins with cold-responsive phosphopeptides within 10 min of cold stress according to KEGG pathway analysis (Figure 4D). In detail, dozens of proteins concerning PtdIns signaling were identified with cold-responsive phosphopeptides, including PtdIns phosphate kinases, PIs phosphatases, phospholipases, phosphatidate phosphatases, PtdIns binding proteins, PIs binding proteins, inositol hexakisphosphate (InsP6) binding proteins and PA binding proteins (Figure S6A). Within proteins involved in PIs biosynthesis and degradation [43], eight proteins contain cold-regulated phosphopeptides (Figure S6B), suggesting that protein phosphorylation play extensive roles in the cold-induced regulation of PIs levels.

As a well-established signaling phospholipid, PA has been demonstrated to possess extensive and multi-regulatory functions, and the PA content was significantly increased upon freezing stress in *Arabidopsis* [41,44]. It was found that different PA molecular species produced by different isoenzymes could function oppositely upon the same stress, for instance, Phospholipases D δ (PLDδ) plays positive role while PLDα1 plays negative role in *Arabidopsis* freezing tolerance [45,46], which suggests the complicated and tight regulation of PA level. PLD-mediated PA production is considered as one of the primary routes of PA signaling, and here we identified three family members (PLDγ1, PLDζ1 and PLDζ2) containing cold-responsive phosphopeptides (Table S5 and Figure S6A). Moreover, two other phospholipases PLDβ1 and Phospholipases C 2 (PLC2), and one lipid phosphate phosphatase LPP1, which is a PA-removing enzyme, responded to cold stress at phosphopeptide levels even though to a lesser degree (Table S6). Therefore, (de)phosphorylation of these proteins might contribute to the rapid PA increase upon cold stress as reported previously [41,47]. In addition to affecting membrane property and serving as precursors for other signaling molecules, PA functions in signaling and growth by binding numerous proteins and regulating their activity and/or subcellular localization. In this study, by searching more than 80 PA targets (or candidates) [44,48,49,50], we found that nine of them contain cold-regulated phosphopeptides (Figure S6A), and could identify six more if we set less stringent cutoffs (fold change > 1.5 or <0.67 and adjusted *p* value < 0.05) (Table S6). These proteins were demonstrated as crucial factors in biological processes including PIs metabolism (Phosphatidylinositol 4-kinase alpha 1 (PI4KA1) and Phosphatase and tensin homolog deleted on chromosome ten 2A (PTEN2A)), osmotic stress (SnRK2.4 and SnRK2.10), ethylene signaling (Constitutive triple response 1 (CTR1)), ABA-mediated reactive oxygen species (ROS) production (Respiratory burst oxidase homolog D (RBOHD)), cortical microtubule polymerization (Microtubule-associated proteins 65-1 (MAP65-1)), MAPK cascade signaling (MPK6) and tricarboxylic acid (TCA) cycle (Phosphoenolpyruvate carboxylase 3 (PPC3)) (Tables S5 and S6 and Figure S6A). In addition, the PA target protein Low expression of osmotically responsive genes 2 (LOS2) is a bifunctional enolase that was demonstrated to regulate cold-responsive gene transcription [51] (Table S6). Besides, the crucial cold signaling protein Sap and miz1 domain-containing ligase 1 (SIZ1), which directly binds to PA in vitro [52], contains three cold-responsive phosphopeptides in our results (Table S6). These results expanded our knowledge of phosphorylation-mediated regulation of phospholipid signaling upon cold stress.

### 2.7. Cold-Mediated Phosphorylation Modification in Cytoskeleton Proteins

The cytoskeleton is a highly dynamic structure which play essential roles in many biological processes including signal transduction, and it was supposed to contribute to the Ca^2+^ influx upon cold shock [53]. The Ca^2+^ influx response, which occurred almost simultaneously with the onset of cold treatment [22], was found to significantly enhanced by microtubule disorganization, and microfilament depolymerization showed limited effects in this process [54]. Here, in our data, GO analysis indicated that proteins concerning cytoskeleton were enriched among the proteins with cold-responsive phosphopeptides (Figure S7A), suggesting important roles of phosphorylation plays in cytoskeleton mediated cold response. For instance, many representative microtubule-associated proteins (MAPs) and actin binding proteins (ABPs) involved in plant response to environmental signals [55] contain cold regulated phosphopeptides (Figure S7B). MAP65-1, responsible for binding, bundling and stabilizing microtubules, phosphorylated at T552 in a microtubule-interacting region (Table S9), a phosphorylation of which could disrupt the interaction [56]. It was reported that MAP65-1 is phosphorylated by its upstream kinase MPK4 and MPK6 [57], and MAP65-1 was also found to bind to PA and function in linking up membrane lipids and the cytoskeleton in environmental stress signaling [58]. In addition, a Ca^2+^-regulated protein Villin 3 (VLN3), which is involved in actin filament bundling, is speculated to serve as a common hub in stress signaling as it can be phosphorylated by multiple stress-activated kinases [37]; here, we demonstrated that three phosphopeptides (S826, T840 and S773) of VLN3 were downregulated upon cold stress, and its upstream kinases MPK6 and SnRK2.4 contain cold-responsive phosphopeptides (Table 2, Tables S5, S6 and S9 and Figure S7B). These results expanded our knowledge concerning phosphorylation modification in the regulation of plant cytoskeleton mediated cold response.

### 2.8. Cold-Mediated Protein Phosphorylation in Calcium Signaling Proteins

As a universal second messenger, Ca^2+^ is involved in signal transduction in various biological processes including cold stress. Plants perceive cold signals and activate Ca^2+^ permeable channels to induce transient increase of cytosolic Ca^2+^ concentration, referred to as a Ca^2+^ signal, and it is crucial for downstream physiological responses. Here, we identified cold-responsive phosphopeptides representing a number of proteins involved in Ca^2+^ influx system, including the cyclic nucleotide gated channel family protein CNGC5, the ionotropic glutamate receptor family protein GLR3.5, the mechanosensitive-like channels (MSL5, MSL9, and MSL10), the hyperosmolality-induced [Ca^2+^]_cyt_. channels (OSCA1 and OSCA2.2/ERD4), and mid1-complementing activity channel protein MCA1 (Figure S8 and Table S5). As a Ca^2+^ permeable mechanosensitive channel, MCA1 was demonstrated to mediate cold-induced cytosolic Ca^2+^ increase and cold tolerance in *Arabidopsis* [59], and here, MCA1 was rapidly dephosphorylated at S278 within 10 min of cold shock, suggesting important role of phosphorylation modification plays in this process (Table 2 and Figure S8). Recently, the Ca^2+^ permeable transporters Annexin1 (ANN1) and ANN4 were found to participate in cold-induced cytosolic Ca^2+^ increase and freezing tolerance, and phosphorylation of ANN1 at S289 site by OST1 is required in these processes [60]. In our data, phosphorylation of S289 in ANN1 was not significantly increased within 120 min of cold treatment; instead, phosphorylation of S46 and S48 in ANN4 was increased (Table 2 and Table S9). After the transient increase of cytosolic Ca^2+^ concentration, subsequent reestablishment of the concentration to resting levels is of crucial important for the correct Ca^2+^ signal transduction, and the extrusion system including Ca^2+^ pumps and Ca^2+^/H^+^ exchangers (CAXs) are required. In our data, we identified four Ca^2+^-transporting ATPase (ACA4, ACA8, ACA9 and ACA10) and one calcium/cation exchanger protein CAX1, which contains cold-responsive phosphopeptides, and only the phosphopeptide of ACA10 was up-accumulated, while the others were down-accumulated (Figure S8 and Table S5). *CAX1*, a cold inducible gene, was demonstrated to play negative roles in the cold acclimation in *Arabidopsis* [61], and here, we give evidence that phosphorylation regulation may participate in this process (Figure S8 and Table S5). To sum up, our findings indicate that the cytosol Ca^2+^ transient increase upon cold stress might be regulated by protein (de)phosphorylation of Ca^2+^ permeable channels and transporters.

The Ca^2+^ sensors, including calmodulins (CaMs), CaM-like proteins (CMLs), calcineurin B-like proteins (CBLs), and CPKs, decode the cold-triggered Ca^2+^ signals to the downstream signaling events [3]. In this study, we identified cold-responsive phosphopeptides representing over 60 proteins involved in the Ca^2+^ signal sense and transduction, including five CPKs (CPK1, CPK5, CPK6, CPK13, and CPK28), four CaM-binding transcription activator family proteins (CAMTA1, CAMTA2, CAMTA3, and CAMTA5), four CBL-interacting protein kinases (CIPK8, CIPK9, CIPK10,CIPK12), 15 calcium-dependent lipid-binding (CaLB domain) family proteins, 16 other calmodulin-binding proteins, and 12 other EF-hand containing proteins (Figure S8 and Table S5). Among the six members of the CAMTA family in *Arabidopsis*, four of them were demonstrated as essential factors contributing to cold response. *CAMTA3* and *CAMTA5* function in regulating the expression of *CBF1* and *CBF2* in response to a rapid decrease of temperature [62], and other studies found that *CAMTA1-3* function together to induce *CBF1-3* expression and enhance freezing tolerance [63]. However, expression of all these four *CAMTAs* are not induced by cold [62], indicating that cold regulates these genes by post-transcriptional regulation, and here, we provided evidence that CAMTA1-3 and CAMTA5 are regulated by cold at phosphopeptide levels (Figure S8 and Table S5). In addition, three CPKs in rice, OsCPK17, OsCDPK13 and OsCDPK7, were shown to function in cold tolerance [64,65,66], and our study found that their *Arabidopsis* orthologs, CPK1, CPK5 and CPK6, are regulated by cold at protein phosphorylation levels (Figure S8 and Table S5). These results suggested that protein phosphorylation mediated Ca^2+^ signal perception and transduction play vital roles in the early cold response.

### 2.9. MAPK Cascades Response upon Cold Stress

In this study, we identified 146 kinases, which were modified by protein phosphorylation by cold, about one-fourth of the kinases are involved in MAPK cascades. MAPK cascades have been demonstrated to play vital roles in converting environmental signals into cellular responses. In *Arabidopsis*, a model for the MAPK cascades mediated cold signal transduction has been proposed, cold activated MPK3 and MPK6 negatively regulate cold response by phosphorylate and destabilize ICE1 [8,9], whereas the MEKK1-MKK2-MPK4 cascade, two Ca^2+^/CaM-regulated receptor-like kinase CRLK1 and CRLK2 and a MAPKKK YDA are all found to suppress the cold activation of MPK3 and MPK6 [8]. Of the 120 family members of the MAPK cascade kinases in *Arabidopsis*, 76 of the 120 kinases were detected, and nearly 50% (37) of them were regulated by cold at phosphorylation levels, including six MAPKs, two MAP kinase kinase (MKKs), 25 MAP kinase kinase kinase (MAPKKKs or MEKKs) and four MAP kinase kinase kinase kinase (MAP4Ks) (Figure S9 and Table S5). In addition, phosphorylation of MPK3 (Y198), MPK4 (T201 and Y203) and MPK6 (T221 and Y223) were significantly increased upon cold stress, consistent with a previous proteomics study [8] (Table 2, Tables S6 and S9). By comparing with phosphorylation modification data of the research, which focused on cold-responsive MAPK cascades [8], we found that 16 of the 18 p-sites were identified in our research, and most of them showed similar cold-responsive profiles as reported, in addition, 37 more sites were detected in these kinases (Figure S9), suggesting the comprehensiveness of our phosphoproteomic data. However, three phosphopeptides showed inconsistent cold-responsive patterns, the previous report indicated that Y189 of MPK16 and Y189 of MPK18 did not respond to cold stress and phosphorylation of S65 of MKK2 significantly increased upon cold stress [8], but our data showed that phosphopeptides (Y189) of the two kinases were upregulated, and phosphopeptide of MKK2 (S65) was not significantly changed, instead a phosphopeptide shared between MKK1 and MKK2 (FLTQSGT(Phospho)FKDGDLR) was downregulated upon cold stress (Table 2 and Figure S9). The activity of MKK2 was strongly activated by cold, but the specific role of this kinase in regulating cold response was still controversial, since it was found to positively regulate freezing tolerance by activating MPK4 and MPK6 [67], while another report demonstrated that *mkk2* mutant did not have reduced freezing tolerance although the MPK4 activity was blocked [8]. In addition, phosphopeptides belonging to MKK4 (S344), one of the upstream kinases of MPK3 and MPK6 [68], were significantly increased upon cold stress (Table 2 and Figure S9). Taken together, the MAPK cascades that mediate cold signal transduction are far more complicated and merit in-depth further study.

### 2.10. Phosphorylation Modification of Transcription Factors in Early Cold Response

More than 1700 loci in the *Arabidopsis* genome encode transcription factors (PlantTFDB v5.0: http://planttfdb.gao-lab.org/index.php?sp=Ath, accessed on 20 November 2021) [69], and approximately one-third of them were found to be cold-responsive genes by transcriptomic studies [70,71,72,73], indicating the extensive transcription regulation in response to cold stress. Here, we found that 435 transcription factors are phosphoproteins in *Arabidopsis* seedlings, and 10% (43) of them contain cold-responsive phosphopeptides (Figure S10), including nine members of C3H family, eight members of bZIP family and four members CAMTA family. Besides, of the 43 cold-responsive proteins classified as transcription factors, 19 of them were also cold-responsive at transcription levels during longer times of cold treatment, further supporting their roles in cold tolerance.

The typical transcriptional regulation of cold response includes CBF-dependent and -independent pathways, and many transcription factors contribute to the transcriptional regulation of *CBFs*, including ICE1, CAMTAs, Myb domain protein 15 (MYB15), Circadian clock associated 1 (CCA1), Late elongated hypocotyl 1 (LHY), Brassinazole resistant 1 (BZR1), BRI1-EMS-suppressor 1 (BES1), CESTA (CES), Ethylene insensitive 3 (EIN3), and Phytochrome interacting factors (PIFs) [3,4]. Many other transcription factors, which are rapidly induced by cold in parallel with *CBFs* and confer transcription regulation of a set of *CORs*, also play important roles in the cold response, such as *Heat shock transcription factor c1 (HSFC1)*, *ZAT12*, *ZAT10*, *CZF1*, *Related to ABI3*/*VP1 1* (*RAV1*), *Tandem zinc finger 3* (*TZF3*) [71]. Previous reports found that the cold-induced *CBFs* expression is gated by the circadian clock [74], and CCA1 and LHY, the two core components of the clock, were found to respond to cold stress at protein phosphorylation level, as two phosphopeptides of CCA1 (S110 and S475) were only detected in samples with cold treatments of more than 30 min, while phosphopeptides of LHY (T404) were only detected in the control samples (Table S7). Besides, we uncovered increased phosphorylation levels at S157 in MYB-like transcription factor REVEILLE 8 (RVE8), which is a circadian clock protein and regulates plant thermotolerance [23] (Table 2 and Figure S10). These results suggest phosphorylation regulates function of transcriptional factors involved in crosstalk between circadian clock and cold signaling. The transcription factor Elongated hypocotyl 5 (HY5) and its suppressor E3 ubiquitin ligase Constitutive photomorphogenic 1 (COP1), two central hub proteins in photomorphogenesis [75,76], were involved in cold acclimation in *Arabidopsis* [77], and here we found that protein phosphorylation might play roles in this process, as phosphopeptides of HY5 (S36) and COP1 (T6) were cold-responsive (Tables S6 and S7). Phytochrome B (phyB), a phytochrome that also serves as a thermosensor [78,79], plays positive roles in freezing tolerance, and its stability is regulated by the interaction between CBFs and PIF3 under cold conditions [80], here in this study, we demonstrated that two phosphopeptides of phyB (S86 and T1159) were significantly altered upon cold stress (Tables S5 and S6), suggesting that the CBFs-PIF3-phyB module in cold response involves protein phosphorylation regulation. In addition, other transcription factors which are induced by cold in parallel with *CBFs* [71], such as RAV1 and TZF3, also contain cold-responsive phosphopeptides (Tables S6 and S7).

The key transcription factor ICE1 in cold signal transduction regulates the transcription of both *CBFs* and *CORs* [81], and the activity and stabilization of ICE1 is regulated mainly at post-translation level. Here, we found that the phosphorylation of S403 of ICE1, a key residue that plays roles in the attenuation of cold response by regulating protein stabilization [82], was not significantly altered within 120 min of cold stress; instead, a peptide containing phosphorylated S172 was only detected in cold-treated samples, and the maximum abundance was reached at 30 min of cold stress (Table 2 and Table S7), suggesting that phosphorylation modification of different sites of ICE1 might contribute to cold response at different stages. Moreover, our data uncovered cold-upregulated phosphopeptides belonging to SIZ1 and High expression of osmotically responsive genes 1 (HOS1) (two crucial proteins involved in ICE1 abundance regulation), and OST1 and MPK3/6 (upstream kinases of ICE1) (Table 2, Tables S6 and S9). These data support the extensive and complicated regulation of ICE1 in the cold response process.

### 2.11. Phosphorylation Modification of Proteins Belonging to COR Genes in Early Cold Response

To investigate whether protein phosphorylation plays roles, or to what extent, in the *COR* gene function regulation, we collected cold-responsive genes from four publications [70,71,72,73], and totally obtained 7189 *COR* genes. We found that 28% (1981) of the CORs are phosphoproteins, and 5% (348) contain cold-responsive phosphopeptides (Figure S11A). Approximately 8% (555) of the CORs are identified as CBF regulons, and here we demonstrated that 4% (24) of the regulons contain cold-responsive phosphopeptides, and only four appeared in all four datasets, they are RD29A with up-accumulated phosphopeptides and three late embryogenesis abundant (LEA) proteins (COR47, Early response to dehydration 10 (ERD10), and AT2G23120) with down-accumulated phosphopeptides upon cold stress (Table 2, Table S9 and Figure S11C). These data suggested that many important CORs respond to cold by both transcription and protein phosphorylation regulation.

COR47, ERD10 and ERD14, three acidic subclass dehydrins (group II LEA proteins), showed up-accumulated protein abundance and phosphorylation level upon cold stress in the previous reports [83]. However, in this study, the cold-responsive phosphopeptides of these three dehydrins were all down-accumulated (Figure S11B), and it may be explained by the different cold stress time (within 2 h versus 2 days) and the different tissue materials used. All three dehydrins have subcellular localization at cytosol and could interact with the plasma membrane [84], and they are all shown to have phosphorylation-dependent Ca^2+^ binding activity [83], suggesting their potential contribution in signal transduction. Moreover, in response to osmotic stress, EDR10 and ERD14 are found to be phosphorylated by the ABA-non-activated SnRK2.10 [85], and here we found that the phosphopeptides (S154 and S354) of SnRK2.10 were initially decreased, and then increased, and finally decreased again upon cold stress (Table S6), indicating a potential ABA-independent SnRK2s-dehydrins process in regulating early cold response.

### 2.12. Validation of Cold-Induced Protein Phosphorylation Level Changes

**Figure 5:**
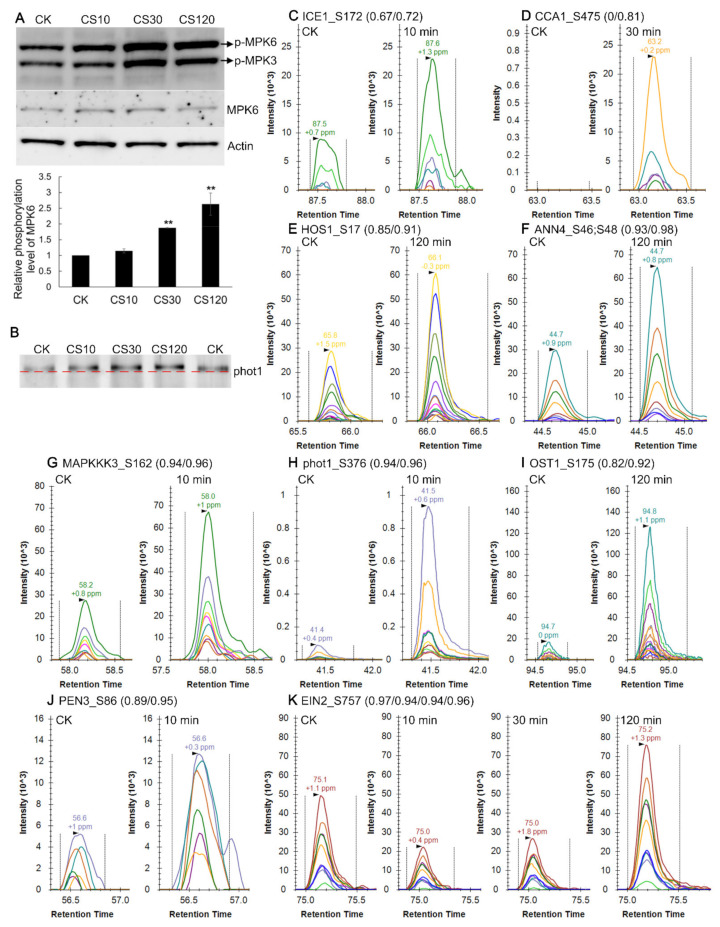
Validation of protein phosphorylation alteration upon cold stimuli by Western blot (**A**,**B**) and targeted mass spectrometry analysis (**C**–**K**). (**A**,**B**) Arabidopsis seedlings were cold treated for 10, 30, and 120 min. Total proteins of seedling samples were immunoblotted using anti-pTEpY, anti-MPK6, anti-Actin and anti-phot1, respectively, the histogram shows the relative phosphorylation level of MPK6 (normalized to total MPK6 protein abundance). (**C**–**K**) Representative extracted ion chromatograms (n > 5 co-eluting fragments) are shown for cold-modified p-sites for (**C**) ICE1:S172, (**D**) CCA1:S475, (**E**) HOS1:S17; (**F**)ANN4:S46/S48; (**G**)MAPKKK3:S162; (**H**) phot1:S376; (**I**) OST1:S175; (**J**) PEN3:S86; (**K**) EIN2:S757. The peak correlation to the library spectra (dotp) is indicated in parentheses after the p-sites of each protein. Three or four biological repeats were performed for each PRM validation, and the p-sites assignments were manually checked. ** *p* < 0.01.

Cold-responsive phosphorylation of selected proteins was further validated by Western blot and PRM analyses. Immunoblotting using anti-pTEpY, which recognizes phosphorylated MPK3, MPK4, MAP6 and MPK11 proteins [8], showed that phosphorylated MPK3 and MPK6 increased significantly after 30/120 min exposure (Figure 5A), in agreement with a previous study [8]. Immunoblotting using anti-MPK6 antibody showed that the protein abundance of MPK6 did not change upon cold shock, indicating increased phosphorylated MPK6 proteins resulted in higher phosphorylation stoichiometry level (Figure 5A). In addition, the blue light receptor phototropin 1 (phot1) protein exhibited slower electrophoretic mobility upon exposure to cold, in as early as 10 min (Figure 5B), supporting that phot1 is rapidly phosphorylated by cold shock.

Because of limited available antibodies, PRM was used to validate findings from DIA studies [86]. Our PRM assays of the cold-responsive proteins were consistent with the DIA results, using tubulin beta-5 chain protein as the internal loading control (Table 1). For the validation of cold-responsive phosphopeptides, phosphopeptides from kinases (especially those implicated in the MAPK signaling cascades), transcriptional factors, E3 ligases, phosphatases, and those discussed previously were chosen for PRM analysis. In this study, 89 cold-responsive phosphopeptides (from 68 proteins) were verified by PRM which showed similar response profiles with DIA data, and 73 (from 62 proteins) of them met the cutoff of fold change >2 or <0.5 (*p* value of *t* test <0.05) in at least one time-point of cold treatment (Table S9), and results at 120-min time point were listed in Table 2.

In the MAPK cascades, 23 cold-responsive phosphopeptides were validated, including MPK3, MPK4, and MPK6 (Table 2). We confirmed the increased phosphorylation of MPK18 (Y189) after 30 min of cold shock (Table 2 and Table S9). Increased phosphorylation of MAPKKK3 (S162), YDA (S794), MAP4K5 (S352), and alteration of phosphorylation levels of a number of RAFs were also validated by PRM (Table 2 and Table S9). We confirmed the decreased phosphorylation level of T161 at Cell division control 2 (CDC2) (Table 2), phosphorylation of which was known to be required for CDC2 function in cell division during male gametogenesis [87]. Phosphopeptide levels of Phosphatase and tensin homolog deleted on chromosome ten 3 (PEN3, S86), which belongs to the dual phosphatase family with both protein tyrosine phosphatase and phosphoinositide phosphatase activity [88], were significantly increased within 10 min of cold shock (Figure 5 and Table S9). Besides, increased phosphorylation of HOS1 (S17) upon cold stress within 2 h was validated (Figure 5 and Table S9). Therefore, the consistent results between DIA and PRM indicate the reliability of the identified cold-responsive phosphorylation events in this study.

The validated phosphopeptides include 31 phosphopeptides with complete missing values in the four biological repeats of either control or 2 h-treated samples. Most of such phosphopeptides with complete missing values in either time point in DIA analysis, were correctly identified and quantified by PRM, except that signals of the CCA1 phosphopeptides (S475) and RAF13 (S465 and S468), were completely below detection in the control samples and 2 h-treated samples, respectively. PRM assays showed these phosphopeptides exhibited lower signals in samples with complete missing values than in samples without any missing values in DIA analysis, as expected. For instance, cold stress increased levels of phosphopeptides, representing a number of transcription factors, including ICE1 (S172), Response regulator 1 (ARR1, S374) and ARR2 (S12), all with missing values in the control sample groups in DIA analysis (Table 2). In addition, decreased phosphopeptide levels were observed for Hercules receptor kinase 2 (HERK2, T498) and Plant u-box 4 (PUB4, S492), from DIA results with missing values in cold-treated sample groups (120 min) (Table 2). Since the major difference between PRM and DIA is the much narrow precursor windows (1.2 *m*/*z* in PRM vs 24 *m*/*z* or larger in DIA in our analysis), suggesting that these missing values were caused by the poorer quality of the product ions which could not meet the criterion of the DIA-NN algorithm, likely resulting from the interference from other co-isolating ions with higher signals in the samples or in the matrix.

In the time-course experiment of cold treatment, some proteins showed dynamic changes at the phosphorylation levels, such as Ethylene insensitive 2 (EIN2). DIA analysis showed that four phosphopeptides within the C-terminal domain of EIN2, containing phosphorylated S645, S757, S924 and S1283, respectively, were initially decreased (within 30 min) and then increased (120 min) upon cold stress (Table S5), and PRM validated phosphorylation levels of S645 and S757 followed the expected down- and then upregulation pattern (Figure 5 and Table S9). These p-sites correspond to the four reported CTR1 targets [89], and their dephosphorylation were known to relay signal to the downstream transcriptional cascades, suggesting a rapid ethylene response and afterwards attenuation during cold stress.

## 3. Discussion

**Figure 6:**
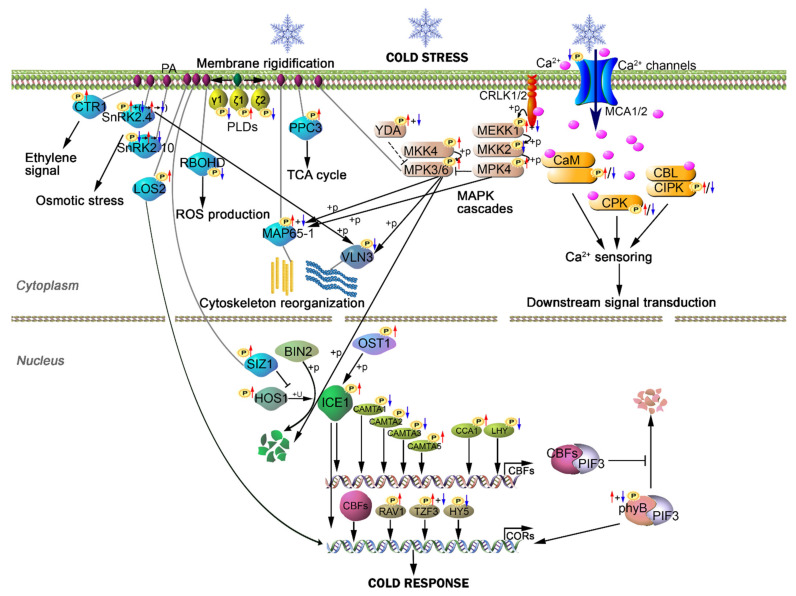
Schematic model illustrating protein phosphorylation and dephosphorylation events during early cold response in *Arabidopsis* based on our phosphoproteome study. At the onset of cold treatment, the cell membrane rigidification, cytoskeleton reorganization and cytosolic Ca^2+^ increasing occur rapidly, followed by activation of cold signal transduction pathways such as Ca^2+^ signal relay and MAPK cascades, and then transcription factors respond to cold signal and transcription regulation occurs. MCA1 is a Ca^2+^ permeable mechanosensitive channel which contributes to the cold-induced transient cytosolic Ca^2+^ increasing. CaMs, CBLs, CIPKs and CPKs decode and relay Ca^2+^ signals to the downstream signal transduction. The lipid-derived PA serve as important signaling phospholipids and have multi-regulatory functions by binding proteins involved in various biological processes. MAP65-1 and VLN3 function in linking up cytoskeleton with other signal pathways including phospholipid signal and MAPK cascades. The key transcription factor ICE1 in cold signal transduction is mainly regulated by cold at post translational modification level. OST1 phosphorylates and activates ICE1, while MPK3/6 and BIN2 phosphorylate ICE1 and promote its degradation. HOS1 ubiquitinates ICE1 and mediates its degradation while SIZ1-mediated sumoylation of ICE1 inhibits this process. The cold-induced crucial transcription factors CBFs are transcriptionally regulated by many transcription factors, including ICE1, CaM-binding transcription activator family proteins (CAMTAs) and core components of circadian clock (CCA1 and LHY). In addition to *CBFs*, *CORs* gene expression are directly regulated by a number of transcription factors, such as RAV1, TZF3, HY5, and also ICE1. CBFs contribute to the stability regulation of phyB, a phytochrome which serves as thermosensor, by interacting with PIF3. Black arrows denote activation while lines ending with a bar indicate inhibition; gray lines denote interaction between PA and its target proteins; +P and +U beside the black arrows denote phosphorylation and ubiquitination events respectively; red up arrows beside the small P icons which are surrounded by yellow circles indicate upregulated phosphopeptides while the blue down arrows indicate downregulated ones identified in this study.

In recent years, significant progress has been made in the dissection of the molecular mechanisms of cold tolerance; however, most studies were focused on the ICE1-CBF-COR pathway, which controls the expression of a relatively small percentage of *CORs* upon cold stress [72,73,90]. Studies on ICE1-independent and/or CBF-independent pathways are still limited. Besides, the early events of signal sense and transduction in response to cold, which mainly occur on post-translation level, are largely unknown. In this study, a comprehensive time-course profiling of proteome and phosphoproteome of *Arabidopsis* seedlings upon cold stress were performed. Our results indicated that, upon short periods of cold treatments, the phosphoproteome of *Arabidopsis* seedlings was significantly altered, whereas the global proteome was largely unaffected. Based on our phosphoproteomic data and genetic advances in cold tolerance in *Arabidopsis*, we proposed a model describing the potential (de)phosphorylation events in *Arabidopsis* seedlings upon cold stress within 2 h (Figure 6), including the signaling pathways of phospholipids, cytoskeleton, calcium signal, MAPK cascades and transcription regulation.

In this study, a comprehensive *Arabidopsis* phosphoproteome library generated by Spectronaut (ProteomeXchange identifiers PXD028188), with over 24,000 high confidence p-sites, each with indexed retention time (iRT) information, provides a complementary resource for the *Arabidopsis* phosphoproteome community. Comparison of the high-confidence p-sites identified in this study with three other recent large-scale phosphoproteomics studies in *Arabidopsis* [27,30,91] found that a quarter of the high-confidence p-sites in our phosphoproteome were uniquely identified compared to other reports (Figure S12 and Table S2).

Our study provides a rich phosphoproteomics resource for studying cold response in *Arabidopsis*, including over 2000 cold-responsive phosphopeptides. Moreover, our data support previous genetic, biochemical and proteomics studies. For instance, of the major known proteins involved in cold signal transduction in *Arabidopsis* [3], most were found to be rapidly modified at phosphorylation levels in our study, including ICE1, HOS1, SIZ1, OST1, CAMTAs, phyB, MPK3/4/6, MKK2/4, YDA, MEKK1, CPKs and CIPKs (Figure 6 and Tables S5–S7). Besides, among the 18 p-sites of MAPK cascade proteins reported previously in a phosphoproteomics work [8], 16 of them were identified in our study and 13 p-sites showed similar cold-response profiles (Figure S9), except that three phosphopeptides showed somehow different cold-response patterns, likely because of different plant growth and treatment conditions. In addition, using PRM assay, we validated 26 cold-responsive p-sites representing MAP kinase cascade components (including RAFs) (Table 2), 19 of which were not reported in the previous study [8]. Therefore, our results strongly support the powerfulness of DIA-MS based quantitative phosphoproteomics in uncovering early phosphorylation/dephosphorylation events during rapid signal transduction.

Our study also provides a phosphoproteomics resource for studying the interaction of cold response with other signaling events in plants. Of the early cold-responsive phosphoproteins, many are known to be involved in hormonal or environmental signaling events, including blue light response and ABA signaling. Here we identified a large number of p-sites at blue light receptors phot1 and phot2, and PRM analysis validated cold-induced phosphorylation of S350, S376 and dephosphorylation of S382 in phot1, and phosphorylation of S22 in phot2 (Table 2 and Table S9). Phosphorylation of S376 in phot1 was detected only in blue light-treated seedlings [92], while S382 was not detected in both dark and blue light-treated samples, suggesting the complexity of phosphorylation regulation of phot1 by blue light and cold response. ABA signaling is known as an important pathway in plant cold response, and our phosphoproteomics data strongly support that ABA signal was activated upon cold stimuli, as cold-responsive phosphopeptides were identified in proteins of ABA-activated SnRK2s (including SnRK2.2 (S173, S177 and T178), SnRK2.3 (S172 and T177), and SnRK2.7 (T159)) and the downstream transcription factors of ABA-responsive element binding protein 3 (AREB3, S43) and G-box binding factor 4 (GBF4, S158) (Table 2 and Tables S5 and S6). OST1 serves as a key protein involved in both ABA signaling and cold signaling [7,93], phosphorylation of OST1 in the kinase activation loop including S171 and S175 [94,95], were significantly increased upon cold treatments (Table 2 and Table S9), suggesting the importance of S171 and S175 phosphorylation in cold-induced OST1 activation, which is consistent with the recently reported cold-activated p-sites in OST1 [96]. Furthermore, phosphopeptide levels of the ABA receptor PYR1-like 4 (PYL4) (S88) were significantly decreased upon cold stress at the early stage (Table S5), suggesting that S88 might sever as an important phosphorylation site which confer cold-induced ABA receptor activation, as phosphorylation of PYLs on a conserved serine residue which located within ABA binding pocket was found to inhibit PYLs activates under non-stress conditions [91].

In addition to phosphorylation, ubiquitination is another important post-translational modification involved in signal transduction such as cold response. A few key components of cold signaling including ICE1, CBFs and MYB15 are regulated by ubiquitination under cold stress, and protein phosphorylation plays an important role in these pathways [9,97,98]. In this study, we found that 43 E3 ligases contain cold-responsive phosphopeptides (Table S5), and five E3 ligases and four deubiquitinating enzymes (DUBs) contain phosphopeptides only in the control or cold-treated samples (Table S7). The ubiquitin E3 ligase HOS1 and SUMO E3 ligase SIZ1 target and regulate ICE1 abundance in response to low temperature [99,100], and our data indicated that both of them contain cold-responsive phosphopeptides (Table 2 and Table S9). Therefore, our results suggest the comprehensive crosstalk between protein phosphorylation and ubiquitination during cold stress.

We found that dephosphorylation events occur more frequently than phosphorylation upon cold stress, especially within 10 min of cold stimuli (76% of cold-responsive phosphopeptides) (Figure 2H), suggesting rapid phosphatase activation at the early stage of cold response. We found that, at protein phosphorylation level, nine phosphatases (including six PP2C and three PTP family members) responded to cold stimuli (Table S5), and 14 more responded to a lesser extent (Table S6). Using PRM technology, we verified three cold-induced phosphopeptides (Table 2 and Table S9), including phosphopeptides of Map kinase phosphatase 1 (MKP1), a dual-specificity phosphatase that regulates MPKs activities (particularly MPK6) [101,102,103], and C-terminal domain phosphatase-like 1 (CPL1), which is a phosphatase involved in freezing tolerance regulation [104]. Hence, perturbation of phosphatase activities under cold stimuli may also occur at protein phosphorylation level.

Despite the large numbers of phosphoproteomics studies in biomedical sciences, studies on the early signaling events using large-scale phosphoproteomics tools (especially DIA and PRM) are quite limited. Our study uncovers a large number of early cold-responsive phosphorylation/dephosphorylation events in kinases, phosphatases, transcriptional factors, and E3 ligases, further biochemical and genetic studies are required to reveal the biological functions of these events during cold adaption. Our study strongly supports that phosphoproteomics using DIA coupled PRM are valuable in uncovering early phosphorylation/dephosphorylation events in plants during rapid signal response.

## 4. Materials and Methods

### 4.1. Plant Growth and Cold Treatment

The surface-sterilized *Arabidopsis* seeds (ecotype Col-0) were stratified at 4 °C in the dark for 3 days to synchronize germination, and then grown in liquid half-strength Murashige and Skoog medium supplied with 1.5% sucrose for 6 days with shaking at 100 rpm (24 °C; 16/8-h light/dark cycle). The medium was divided into four parts, and three parts were chilled on ice to 2 °C; then, the seedlings were separated into four parts, and three of them were exposed to the cold medium to start cold treatment for 10, 30, and 120 min respectively (CS10, CS30, CS120), and the left one part was used as untreated control (24 °C, CK). In total, 16 samples (four biological replicates of each treatment or control) were harvested and frozen in liquid nitrogen for proteomic and phosphoproteomic analyses.

### 4.2. Protein Extraction and Peptides Preparation

Total proteins were isolated under fully denaturing conditions following a modified protocol [105]. Briefly, approximately 3 g liquid nitrogen-ground tissue powder was thoroughly mixed with 9 mL extraction buffer supplied with phosphatase and protease inhibitors (150 mM Tris-HCl (pH 7.6), 8 M urea, 0.5% SDS, 1.2% Triton X-100, 20 mM EDTA, 20 mM EGTA, 2% polyvinylpolypyrrolidone, 5 mM ascorbic acid, 50 mM NaF,1 mM PMSF, 5 mM DTT, 1% glycerol 2-phosphate, protease and phosphatase inhibitor cocktails (Thermo Scientific, Carlsbad, CA, USA)). The cell debris were removed via centrifugation at 16,000× *g* at 4 °C for 20 min and then proteins were precipitated from the supernatant by mixing with three volumes of cold acetone:methanol (12:1 *v*/*v*) and keeping at −40 °C overnight. The protein pellet was rinsed with cold acetone:methanol:water mix (12:1:1.4, *v*/*v*), redissolved with resuspension buffer (50 mM Tris-HCl (pH 6.8), 8 M urea, 1% SDS, 5 mM DTT, and 10 mM EDTA), and followed by another round of protein precipitation and rinse. The protein pellet was rinsed with cold methanol, air-dried, and then dissolved in 8M urea. The protein concentration was determined with a 2-D Quant Kit (GE Healthcare, Piscataway, NJ, USA) using BSA as a standard.

For trypsin digestion, proteins were reduced using 20 mM Tris(2-carboxyethyl)phosphine hydrochloride (TECP) for 60 min at 30 °C, alkylated with 30 mM iodoacetamide at 25 °C for 40 min in darkness, and then precipitated by mixing with five volumes of cold acetone and keeping at −40 °C overnight. The protein pellet was rinsed one time with cold acetone:methanol:water mix (12:1:1.4, *v*/*v*) and two times with cold methanol, and then air-dried and re-dissolved in 2 mL 0.1 M NH_4_HCO_3_. The protein samples were digested with trypsin (enzyme:substrate = 1:25) for 16 h at 37 °C, and the reaction was terminated by adding trifluoroacetic acid (TFA) to a final concentration of 1%. The resulting peptides were desalted using a Strata X 33 µm Polymeric Reversed Phase column (Phenomenex, Torrance, CA, USA) and resuspended in 0.1% TFA for DIA based nanoLC-MS/MS analysis.

To enrich phosphopeptides, chelating Sepharose Fast Flow slurry (GE Healthcare, Piscataway, NJ, USA) was treated with 0.1 M GaCl_3_ for 1 h with end-over-end rotation and then washed with water, and peptides (from 0.5 mg total proteins) redissolved in 700 μL of 0.1% TFA in 80% ACN were incubated with 40 μL of the GaCl_3_-charged Sepharose slurry for 45 min with end-over-end rotation and followed by three times of wash with 1 mL of 0.1% TFA in 80% ACN. The phosphopeptides were eluted three times with a buffer containing 5% ammonia and 50% ACN and desalted with C18 Stage Tips. The phosphopeptides were dried in a SpeedVac and reconstituted with 0.1% TFA for DIA based nanoLC-MS/MS analysis.

For DDA library construction of total peptides and phosphopeptides, a mixture of 16 peptides or enriched phosphopeptides samples was vacuum-dried and reconstituted in 2% acetonitrile and 5 mM ammonium hydroxide (pH 9–10) and separated on a Waters Acquity BEH C18 with a particle size 1.7 μm (2.1 mm × 100 mm) column using H class UPLC system (Waters, Milford, MA, USA) at a flow rate of 300 μL min^−1^. Peptides were separated by a linear gradient from 2 to 8% buffer B (100% acetonitrile, 5 mM ammonium hydroxide) in 1.5 min, to 24% buffer B in 15.5 min, to 32% buffer B within 4 min, and then from 32% to 70% buffer B in 1 min. The buffer A is 5 mM ammonium hydroxide in water. Total 30 fractions were collected and then combined into 10 fraction pools (two more phosphopeptides fractions were added by redissolving the insoluble phosphopeptides of other fractions). The peptides/phosphopeptides of each fraction were vacuum dried and reconstituted with 0.1% TFA for nanoLC-MS/MS analysis.

### 4.3. Mass Spectrometric Acquisition

Samples were analyzed on an a nanoflow high-performance liquid chromatograph instrument (Ultimate 3000 nano, Thermo Scientific, Waltham, MA, USA) coupled online to a Q Exactive HF (QE-HF) mass spectrometer (Thermo Scientific) equipped with a Nanospray Flex Ion Source (Thermo Scientific). Samples were loaded onto a nanotrap column (100 μm × 2 cm, 5 μm, Thermo Scientific) with a flow rate of 5 µL min^−1^ before washed off into an analytical column (Acclaim PepMap C18, 100 μm × 50 cm, 2 μm, Thermo Scientific) for peptide separation at 40 °C. For the analysis of the global proteomics fractions, peptides were separated by a linear gradient from 5% to 8% buffer B (80% ACN, 0.1% formic acid) in 6 min, from 8% to 20% buffer B in 120 min, from 20% to 40% buffer B in 58 min, and then from 40% to 90% buffer B in 6 min. The buffer A is 0.1% formic acid in water. The flow rate was at 250 nL min^−1^. The LC gradient setting was the same for both DIA and DDA analysis. During the DDA acquisition, the full scan was performed between 350–1500 *m*/*z* at the 120,000 resolution, and the automatic gain control target for the full scan was set to 3E6. The MS/MS scan was operated in Top 20 mode using the following settings: resolution 30,000; automatic gain control target 1E5; maximum injection time: 50 ms; normalized collision energy at 27%; isolation window of 1.4 Th; charge sate exclusion: unassigned, 1, > 6; dynamic exclusion 30 s.

The DIA method consisted of a survey scan at 120,000 resolution from 375 to 1500 *m*/*z* (automatic gain control target of 3E6 or 50 ms injection time). Then, 41 DIA windows were acquired at 30,000 resolution (automatic gain control target 1E6 and auto for injection time). Normalized collision energy was 30%, mass range from 400 to 1200 *m*/*z* with 41 isolation windows (30 windows of 15 *m*/*z*, followed by 6 windows of 25 *m*/*z*, 5 windows of 40 *m*/*z*, with optimized window placement defined in Skyline). The spectra were recorded in profile type.

For the phosphoproteomics fractions, peptides were separated by a linear gradient from 5 to 8% buffer B in 5 min, from 8% to 32% buffer B in 105 min, from 32% to 40% buffer B in 5 min, and then from 40% to 90% buffer B in 5 min. The flow rate was at 250 nL min^−1^. The column oven was set to 40 °C. The LC gradient setting was the same for both DIA and DDA analysis. The DDA acquisition setting of the phosphopeptide fractions was the same as for the global proteomic fraction, with the exception of Top 15 mode with the maximum injection time of 110 ms, which was used for MS/MS scanning. The DIA method consisted of a survey scan at resolution 120,000 from 375 to 1500 *m*/*z* (automatic gain control target of 3E6 or 50 ms injection time), and 30 DIA windows were acquired at 30,000 resolution (25 windows of 24 *m*/*z*, followed by 5 windows of 40 *m*/*z*, 0.5 *m*/*z* overlap).

For all MS samples, standard indexed retention time (iRT) peptides (Biognosys, Switzerland) were included in all the samples for retention time normalization [106].

### 4.4. Mass Spectrometric Raw Data Analysis

For the library construction, the RAW files from phosphopeptides or unenriched peptides were analyzed with the Proteome Discoverer (Version 2.4.0.305) software package (Thermo) using the SequestHT [107] and MS Amanda [108] nodes to obtain comprehensive identification of peptides, since combining results from different search engines would perform better in identifying PSMs than single search engine [109]. The DDA files were searched against Araport11 protein database (total 48,359 entries) with a maximum of 2 missed cleavages, and a minimum peptide length of 7 amino acids. Precursor mass tolerance was set to 10 ppm, with a fragment tolerance of 0.02 Da. Search criteria included carbamidomethylation of cysteine as a fixed modification, oxidation of methionine and acetyl (protein N terminus) as variable modifications. Additionally, phosphorylation of serine, threonine, and tyrosine were allowed as variable modifications for search DDA RAW files from phosphopeptides. Spectral libraries were generated using spectral library generation in Spectronaut 13 from the output of Proteome Discoverer with DDA measurements. Spectral libraries were built from Proteome Discoverer search results using Spectronaut Professional + x64 (13.12.200217.43655) with default settings, with the exception of Best N Fragments per Peptide Max set to 25 instead of 6 for library of phosphopeptides. The peptide library for unenriched peptides or phosphopeptides were exported as tab-separated files for subsequent DIA-NN analysis.

The raw data were processed using DIA-NN (1.7.10 for phosphoproteome and 1.7.11 for the proteome) in high-precision mode with global cross-run normalization enabled. The output was at 1% precursor and protein group levels. Protein quantification was performed using the MaxLFQ algorithm as implemented in the diann R package. Statistical testing was performed using limma package in the R environment (version 3.6.2) at protein levels or phosphopeptides levels. For differential abundance testing between two groups, proteins or phosphopeptides with missing values in the biological repeats were discarded. All protein and phosphopeptide intensity were first log2-transformed. Significant differences were filtered for an average fold-change > 2.0 or <0.5 (at the phosphopeptide level) or an average fold-change > 1.5 or <0.67 (at the protein level), with *p* values adjusted for multiple testing correction by false discovery rate (FDR) (Benjamini–Hochberg < 0.01).

### 4.5. Bioinformatic Analysis

The PCA analysis was calculated using built-in R function prcomp, and visualized using the R package ggplot2, and the log2 transformed intensity values of all peptides/phosphopeptides which shared among 16 samples were used as input data. The HCA analysis of the cold-responsive phosphopeptides was performed on the “Wu Kong” platform (https://www.omicsolution.org/wkomics/main/, accessed on 20 November 2021), and Euclidean distances was used as a distance measurement for row clustering. For motif analysis, 21 amino acids sequence windows, which centered on the phosphorylation sites, were submitted to MotifeR (https://www.omicsolution.org/wukong/motifeR/, accessed on 20 November 2021) and processed using settings as Central amino acid = STY, Width = 10, Minimum number = 20, and *p* value threshold = 0.000001 [35], and the background sequence dataset was generated from Araport11 protein lists. GO enrichment of the proteins with cold-responsive phosphopeptides was performed using an online tool database for annotation, visualization and integrated discovery (DAVID, v6.8) [110] with a background setting as all genome genes, the GO terms enriched with FDR < 0.05 were considered as over-representative terms. KEGG pathway enrichment was performed with the web server tool kegg orthology based annotation system (KOBAS 3.0) [111], and the significantly enriched terms were determined using a criterion of Benjamini and Hochberg corrected *p* value < 0.05. The enriched GO terms and KEGG pathways were visualized using the R package ggplot2.

### 4.6. Immunoblot Analysis

Total proteins (10 μg) were separated by SDS-PAGE (10% acrylamide) and transferred to nitrocellulose membrane (Millipore). For MAPK phosphorylation analysis, the membrane was first immunoblotted with anti-pTEpY (Cell Signaling Technology, Cat#9101; 1:1000 dilution), and then stripped and probed with anti-MPK6 antibody (PhytoAB, Cat# PHY0714, 1:1000 dilution), and finally stripped again and probed with anti-Actin antibody (PhytoAB, Cat# PHY0002, 1:1000 dilution). For phot1 phosphorylation analysis, the transferred membrane was immunoblotted with anti-phot1 (gifts from Dr. Winslow Briggs, 1:1000 dilution). The membrane was then incubated with goat-anti-rabbit IgG-horseradish peroxidase (HRP) secondary antibody before development with ECL SuperSignal West Dura Extended Duration Substrate (Thermo Fisher). The images were obtained using the ImageQuant LAS 4000 mini system (GE Healtcare Life Sciences) and quantified with ImageQuant software (GE Healtcare Life Sciences).

### 4.7. Parallel Reaction Monitoring

Both total peptides (unenriched peptides) and phosphopeptides were analyzed using the same LC-MS/MS conditions. Samples were separated chromatography by Ultimate U3000 nano (Thermo) on a 100 μm × 50 cm C18 column, and then introduced into QE-HF mass spectrometer, as described in the DDA experiments. The LC flow rate was 300 nL min^−1^ and the column temperature was 60 °C. Peptides were eluted over a linear gradient from 5% to 44% buffer B in 89 min, 44 to 90% buffer B in 3 min, and then held at 90% buffer B for 7 min. In PRM experiments, a full mass spectrum was acquired at a mass resolution of 60,000 at *m*/*z* 200 from *m*/*z* 350 to 1250 (AGC target 3E6, 100 ms maximum injection time). Each full scan was followed up 15 fully scheduled, targeted HCD MS/MS scan at a mass resolution of 30,000 at *m*/*z* 200. The MS/MS scan was performed at AGC target 1E5, 60 ms maximum injection time, 27% normalized collision energy and an isolation width 1.2 *m*/*z* units.

Acquired PRM data were analyzed with Skyline 21.1 with manual inspection. This involved peak inspection to ensure accurate selection, integration, and uniformity (in terms of peak shape and retention time) of the peptides. Precursors were considered correctly detected if Skyline autodetected peaks satisfied the following criteria: a library dot product (dotp) > 0.6 and an isotope dot product (idotp) > 0.6, mass error ≤ 5 ppm for precursors and ≤10 ppm for fragment ions; peaks found ratio > 0.5 (with at least 5 fragments detected in three samples). Up to 6 of the most abundant ions found in MS/MS were used for quantification. For phosphopeptide site localization determination, up to 30 of the most abundant ions found in MS/MS were manually checked to assure the confident assignment. Three phosphopeptides (TTFGS(Phospho)QILR from AT5G49890 (Chloride channel protein), VTSIIDSVPES(Phospho)PQRP from AT3G08710 (thioredoxin H-type 9) and LEPVVAKPHS(Phospho)PDNR from AT4G13430 (isopropyl malate isomerase large subunit 1)) that were found to show least variations among the 16 samples in the DIA samples, were used for loading control. For protein verification (Table 1), the unique peptides of each protein used for PRM quantification were: LLKPLNSEYGK for ATCG00710, IQTAAVASPK and NNAGYPHNVVFDEDEIPSGVDVAK for AT1G20340, DNLQEVYFLHPGLQSR for AT4G35750, MQGLTNEGPSASDK for AT3G46640, TPGSSGNSC[+57]PIDALK for AT4G12470, IDC[+57]VPLC[+57]GTR for AT5G14920, DTC[+57]PEC[+57]DGAGFVR for AT5G02160. The peptides used for the loading control is FPGQLNSDLR and GHYTEGAELIDAVLDVVR for AT1G20010 (tubulin beta-5 chain). Isolation lists for PRM analysis of cold-responsive proteins and phosphopeptides were included in Table S10.

## Figures and Tables

**Table 1:** Cold-responsive proteins identified from global proteome study and verified with PRM.

Accession	Description	Fold ^1^	Adjusted *p* Value	Fold	Adjusted *p* Value
**30 min**		**PRM**		**DIA**	
ATCG00710	PSBH, Photosystem II reaction center protein H	0.36	2.3 × 10^−3^	0.35	2.6 × 10^−3^
AT1G20340	PETE2, Cupredoxin superfamily protein	0.43	8.5 × 10^−5^	0.4	3.1 × 10^−3^
**120 min**					
AT1G20340	PETE2, Cupredoxin superfamily protein	0.47	1.9 × 10^−4^	0.37	4.7 × 10^−4^
AT4G35750	SEC14 cytosolic factor family protein	0.42	5.8 × 10^−4^	0.42	2.4 × 10^−6^
ATCG00710	PSBH, Photosystem II reaction center protein H	0.46	4.0 × 10^−3^	0.47	8.2 × 10^−3^
AT3G46640	LUX, Homeodomain-like superfamily protein	0.64	8.0 × 10^−2^	0.65	1.5 × 10^−3^
AT4G12470	AZI1, Azelaic acid induced 1	1.37	2.2 × 10^−3^	1.51	7.4 × 10^−3^
AT5G14920	GASA14, Gibberellin-regulated family protein	1.61	7.1 × 10^−3^	1.83	7.4 × 10^−3^
AT5G02160	FIP, FTSH5 interacting protein	2.02	1.3 × 10^−3^	2.18	4.7 × 10^−4^

^1^ Fold refers to protein abundance ratios of treated sample to control group.

**Table 2:** Representative cold-responsive phosphopeptides validated by PRM (120-min time point).

AGI	Name	Phosphopeptides ^1^	p-Sites	Fold	adj_*p*	Fold	adj_*p*
				PRM Data		DIA Data	
**MAPK cascade kinases**							
AT3G45640	MPK3	ICDFGLARPTSENDFMTEyVVTR	Y198	3.4	1.5 × 10^−2^	1.5	3.8 × 10^−3^
AT4G01370	MPK4	TKSETDFMtEyVVTR^2^	T201;Y203	14.1	7.6 × 10^−5^	5.3	7.3 × 10^−7^
AT4G01370	MPK4	TKSETDFMtEYVVTR^2^	T201	4.8	2.0 × 10^−4^	5.4	4.9 × 10^−8^
AT2G43790	MPK6	VTSESDFMtEyVVTR	T221;Y223	2.3	1.9 × 10^−2^	1.9	2.1 × 10^−1^
AT1G18150	MPK8	AAAAVASTLESEEADNGGGYsAR	S539	0.3	8.7 × 10^−3^	0.4	8.5 × 10^−6^
AT1G18150	MPK8	HHAsLPR	S495	0.5	4.1 × 10^−3^	0.4	1.3 × 10^−4^
AT3G18040	MPK9	SIASLVTtLESPPTSQHEGSDYR	T549	0.4	3.4 × 10^−5^	0.4	2.0 × 10^−5^
AT3G18040	MPK9	SQLtTIYR	T51	0.2	4.3 × 10^−4^	0.3	8.1 × 10^−8^
AT1G53510	MPK18	VAFNDTPTTVFWTDyVATR	Y189	2.6	5.5 × 10^−2^	2.9	2.7 × 10^−6^
AT4G29810	MKK2	FLTQSGtFKDGDLR^2^	T31	0.5	2.0 × 10^−3^	0.5	3.5 × 10^−5^
AT1G51660	MKK4	ASPSQNRsPQNLHQLLPPPRPLSSSSSPTT	S344	2.3	2.5 × 10^−3^	2.4	9.9 × 10^−4^
AT1G53570	MAPKKK3	LSGVVsLESSTGR	S162	3.1	1.3 × 10^−2^	3.9	1.2 × 10^−5^
AT1G63700	YDA	sPGSGGNFYTNSFFQEPSR	S794	2.1	7.4 × 10^−5^	2.5	2.3 × 10^−4^
AT3G13530	MAPKKK7	TPsSVSGNELAR	S452	0.3	2.3 × 10^−2^	0.2	6.4 × 10^−8^
AT4G08500	MEKK1	FKsFDLDK	S119	0.5	1.9 × 10^−3^	0.8	5.3 × 10^−2^
AT4G24100	MAP4K5	GVsAWNFDVR	S352	2.1	2.6 × 10^−4^	2.2	2.7 × 10^−5^
AT5G03730	CTR1	AStFLSSK	T704	12.2	1.7 × 10^−4^	NA in CK	
AT1G08720	EDR1	HNTFLsSK	S823	4.1	1.0 × 10^−4^	NA in CK	
AT3G06620	RAF7	NIGEGAPsWR	S59	0.2	1.8 × 10^−2^	NA in CS120	
AT2G31010	RAF13	KTMsLPsSPHAYR	S465;S468	0.0	2.5 × 10^−1^	NA in CS120	
AT1G79570	RAF20	TNsSLHEFGNK	S130	0.5	1.2 × 10^−2^	0.6	4.3 × 10^−3^
AT2G35050	RAF24	RNtLVtGGVR	T1134;T1137	1.2	6.5 × 10^−1^	1.1	8.9 × 10^−1^
AT5G57610	RAF35	ISGFDGMSsLGQPSYPNPHLQDR	S569	4.9	2.3 × 10^−2^	NA in CK	
**Other kinases**							
AT3G45780	phot1	ALsESTNLHPFMTK	S350	2.1	1.1 × 10^−2^	2.2	1.8 × 10^−4^
AT3G45780	phot1	MsENVVPSGR	S376	10.6	8.4 × 10^−5^	7.4	7.6 × 10^−10^
AT3G45780	phot1	MSENVVPsGR	S382	0.5	1.9 × 10^−2^	0.6	1.9 × 10^−4^
AT5G58140	phot2	sLEIFNPSSGK	S22	2.8	9.1 × 10^−5^	3.9	3.6 × 10^−7^
AT3G50500	SnRK2.2	SSVLHsQPKsTVGTPAYIAPEILLR	S173;S177	14.4	1.5 × 10^−3^	18.9	8.0 × 10^−8^
AT3G50500	SnRK2.2	StVGTPAYIAPEILLR	T178	5.8	1.2 × 10^−4^	9.4	1.2 × 10^−7^
AT4G40010	SnRK2.7	SSVLHsQPK^2^	S154	5.4	1.1 × 10^−4^	4.3	3.6 × 10^−10^
AT4G40010	SnRK2.7	StVGTPAYVAPEVLSR	T159	5.9	5.6 × 10^−4^	NA in CK	
AT4G33950	OST1	sTVGTPAYIAPEVLLK	S175	12.3	1.3 × 10^−6^	NA in CK	
AT4G33950	OST1	SSVLHsQPKsTVGTPAYIAPEVLLK	S171;S175	279.9	1.2 × 10^−5^	NA in CK	
AT2G17290	CPK6	NsLNISMR^2^	S536	2.3	6.1 × 10^−3^	1.7	1.8 × 10^−2^
AT3G53930	ATG1B	SSYGFsVER	S349	2.4	1.1 × 10^−4^	NA in CK	
AT4G21390	B120	NTDTsVVVADLTK	S480	0.1	2.2 × 10^−2^	NA in CS120	
AT4G27300	SD11	NVPDISSsLSLR	S798	0.4	2.8 × 10^−2^	NA in CS120	
AT4G33080	NDR6	KLAFsTVGTPDYIAPEVLLK	S300	5.9	1.1 × 10^−2^	NA in CK	
AT5G28290	NEK3	HRPVDLsANDTSR	S333	0.0	2.4 × 10^−4^	NA in CS120	
AT5G64940	ATH13	SVsIAGIFLPR	S31	0.1	2.1 × 10^−3^	NA in CS120	
AT1G68830	STN7	TVTEtIDEISDGRKtVWWNR	T541;T551	0.2	3.4 × 10^−3^	NA in CS120	
AT2G07180	PBL17	SVtLYEASSDSQGTR	T392	4.2	1.7 × 10^−2^	NA in CK	
AT3G48750	CDC2	TFtHEVVTLWYR	T161	0.5	6.3 × 10^−3^	0.5	4.7 × 10^−3^
AT1G30570	HERK2	LNtLAASTMGR	T498	0.2	1.7 × 10^−3^	NA in CS120	
**Phosphatases**							
AT3G55270	MKP1	YVSKtPLSR	T474	9.4	2.2 × 10^−5^	NA in CK	
AT4G21670	CPL1	DETALPVsSRPTDPR	S839	12.5	3.2 × 10^−2^	NA in CK	
AT3G50110	PEN3	TDDIVPCPPGsSPR	S86	4.9	5.6 × 10^−4^	1.9	1.5 × 10^−5^
**Transcription factors**							
AT2G04880	WRKY1	VVTHNNMLDSEVDDKEGDANKtPQSSTLQSITK	T391	0.1	1.3 × 10^−2^	NA in CS120	
AT2G46830	CCA1	IsSNITDPWK	S475	--	--	NA in CK	
AT3G16857	ARR1	SIFsFDNTK	S374	12.6	1.7 × 10^−2^	NA in CK	
AT3G26744	ICE1	DLSSVPDFLsAR	S172	4.6	1.5 × 10^−2^	NA in CK	
AT4G16110	ARR2	GPDsGTAAGGSNSDPFPANLR	S12	2.7	6.7 × 10^−3^	NA in CK	
AT2G40620	bZIP18	LGSGSGsASDSAGPSAPR	S83	2.4	8.1 × 10^−5^	1.6	2.6 × 10^−3^
AT3G09600	RVE8	VIsPQHELATLR	S157	2.6	7.2 × 10^−4^	2.3	2.8 × 10^−6^
AT4G16150	CAMTA5	ETHEVHAAPAtPGNSYSSSItDHLSPK	T154;T164	14.6	1.9 × 10^−3^	5.0	5.5 × 10^−8^
AT4G16150	CAMTA5	ETHEVHAAPAtPGNSYSSSITDHLSPK	T154	1.9	4.5 × 10^−3^	2.3	9.0 × 10^−6^
**E3 ligases**							
AT2G39810	HOS1	SIsLPTQPNYSSKPVQEALK	S17	2.1	2.8 × 10^−4^	2.3	4.2 × 10^−7^
AT1G67900	NPY8	ALLAAHNIDPSNPNAAAFStTTSIAAPEDR	T549	0.3	3.1 × 10^−3^	NA in CS120	
AT2G23140	PUB4	SGPLAATTsAATR	S492	0.3	4.4 × 10^−2^	NA in CS120	
AT3G08020	PHD finger protein	ATFGSVTQFPAASTsEGNHVDDK	S615	2.1	1.5 × 10^−2^	NA in CK	
AT3G26730	RING/U-boxprotein	NQTQsLsPPDVSR	S428;S430	2.0	2.9 × 10^−2^	NA in CK	
AT3G60080	BTL13	TVsGLGIGMR	S282	0.3	1.0 × 10^−2^	NA in CS120	
AT5G15440	EDL1	RNsLLGGSENGPPPQK	S273	2.1	4.9 × 10^−3^	NA in CK	
AT5G65683	WAVH2	FGFLSNPStPR	T50	0.1	1.2 × 10^−2^	NA in CS120	
**Others**							
AT2G38750	ANN4	KAsKsFFVEDEER	S46;S48	2.2	9.3 × 10^−4^	1.9	1.5 × 10^−4^
AT4G35920	MCA1	STsNVSSGHDLLSR	S278	0.4	6.0 × 10^−4^	0.4	2.6 × 10^−5^
AT5G03280	EIN2	AAPTSNFTVGSDGPPsFR	S645	2.0	8.9 × 10^−3^	2.3	7.0 × 10^−3^
AT5G03280	EIN2	SLSGEGGsGTGSLSR	S655	2.4	1.4 × 10^−3^	2.1	2.9 × 10^−3^
AT5G03280	EIN2	TPGsIDSLYGLQR	S757	1.6	2.3 × 10^−2^	1.7	1.2 × 10^−1^
AT1G20440	COR47	NNVPEHEtPTVATEESPATTTEVTDR	T14	0.3	1.1 × 10^−3^	0.4	1.5 × 10^−3^
AT1G76180	ERD14	KKDETKPEEtPIASEFEQK	T46	0.1	7.0 × 10^−6^	0.2	4.2 × 10^−7^
AT2G23120	LEA protein	AYGAEGHQEPTPGLGGGSTDAPtPSGDAPAATTTDAK	T67	0.4	3.9 × 10^−3^	0.4	6.1 × 10^−5^
AT3G57410	VLN3	AAALAALTSAFNSsSGR	S773	0.4	2.9 × 10^−4^	0.3	1.8 × 10^−2^

^1^ Lowercase indicates p-sites; ^2^ denotes peptides shared by another protein (See Table S9); -- indicates undetected phosphopeptide in control sample groups in PRM assay; “NA in CK” and “NA in CS120” indicate undetected phosphopeptides in control and 120-min cold-shocked sample groups respectively in DIA assay.

## Data Availability

The mass spectrometry proteomics data have been deposited to the ProteomeXchange Consortium (http://proteomecentral.proteomexchange.org, accessed on 20 November 2021) via the iProX partner repository [112] with the dataset identifier PXD028188 for DIA and PRM data, and with the dataset identifier PXD028201 for Raw data of DDA libraries.

## Supplementary Materials

The following are available online at https://www.mdpi.com/article/10.3390/ijms222312856/s1.
