# Supplementary figures and images for: Data-Independent Acquisition-Based Proteome and Phosphoproteome Profiling Reveals Early Protein Phosphorylation and Dephosphorylation Events in Arabidopsis Seedlings upon Cold Exposure
Source: Int J Mol Sci. 2021 Nov 27;22(23):12856. doi: 10.3390/ijms222312856 (PMC8657928; doi:10.3390/ijms222312856)

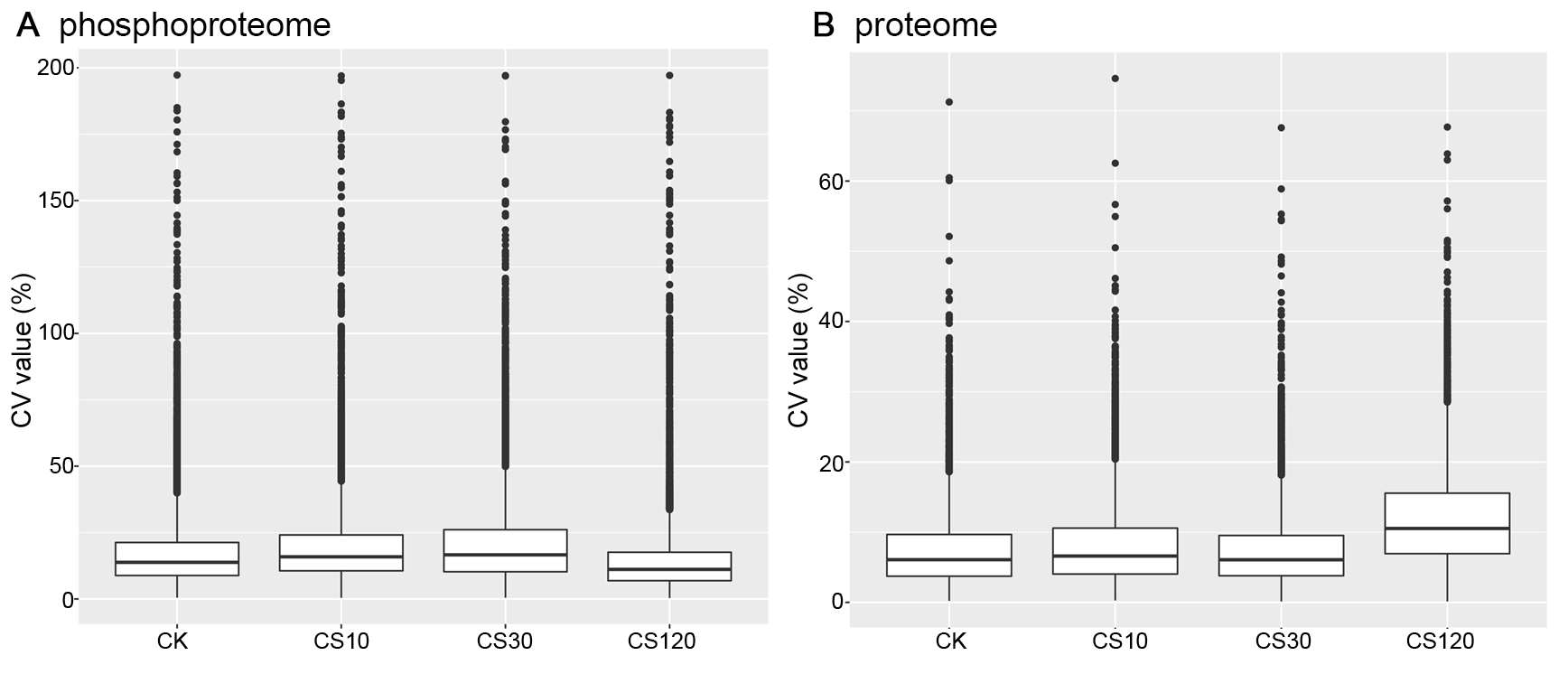

Supplement: Supplementary file 1 [file ijms-22-12856-s001.zip › Figure S1. Box plot representing the between-replicate CV distribution of the quantified phosphopeptides (A) and total proteins (B)..png]

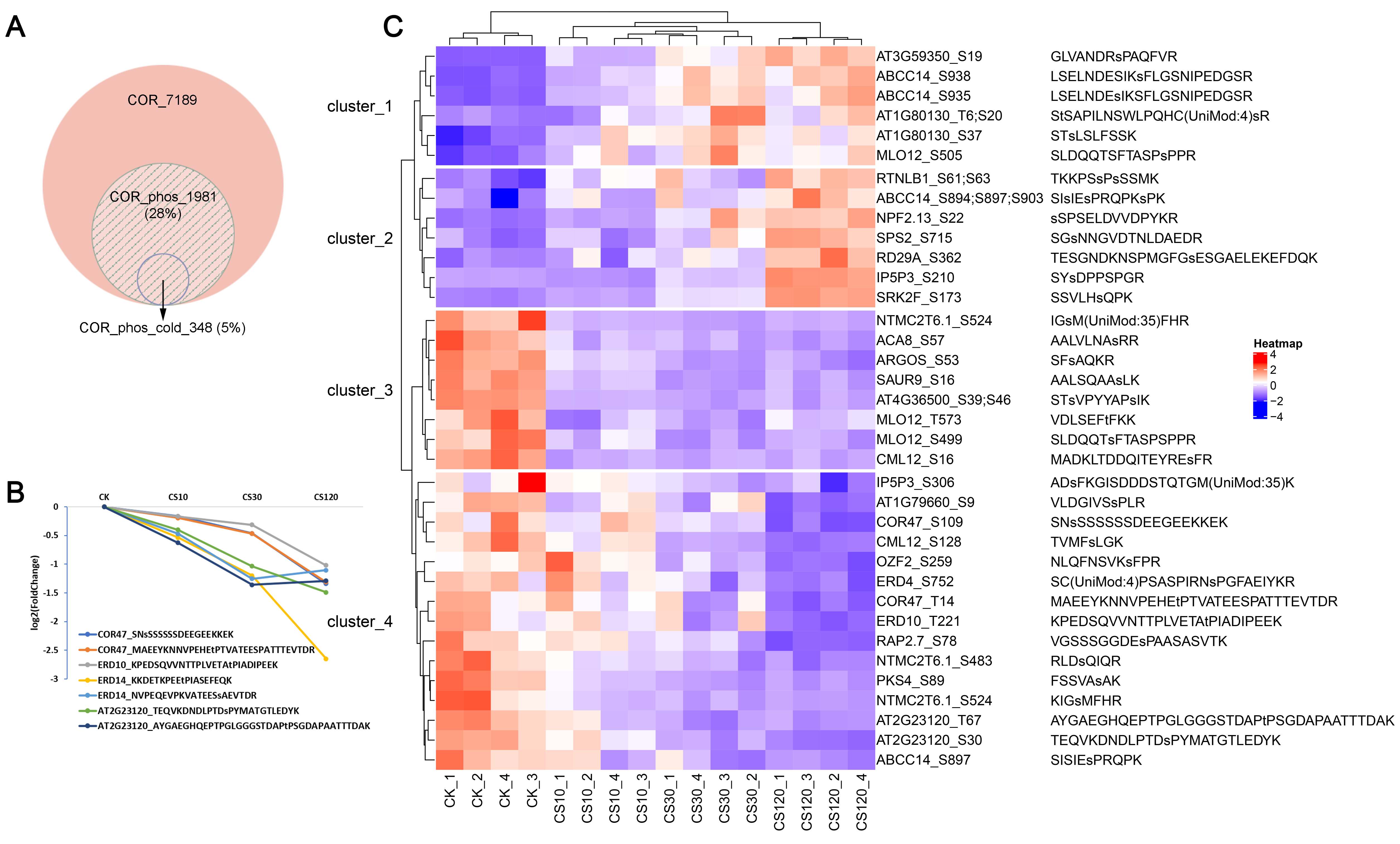

Supplement: Supplementary file 1 [file ijms-22-12856-s001.zip › Figure S11. Cold-Regulated (COR) genes respond to cold stress at protein phosphorylation level.png]

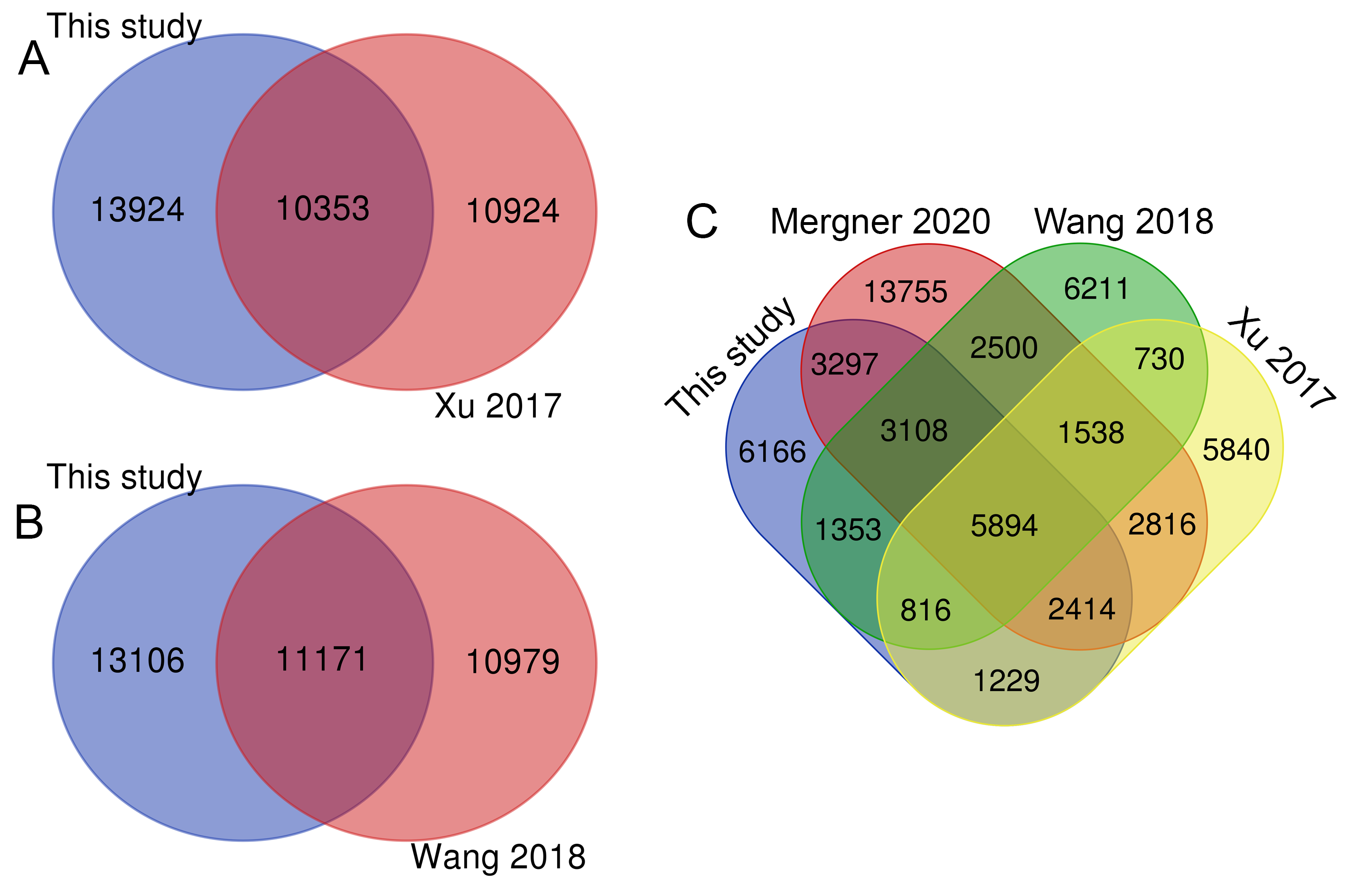

Supplement: Supplementary file 1 [file ijms-22-12856-s001.zip › Figure S12. Venn diagram showing p-sites identified in this study (high-confidence) and three other reported phosphoproteomes in Arabidopsis.png]

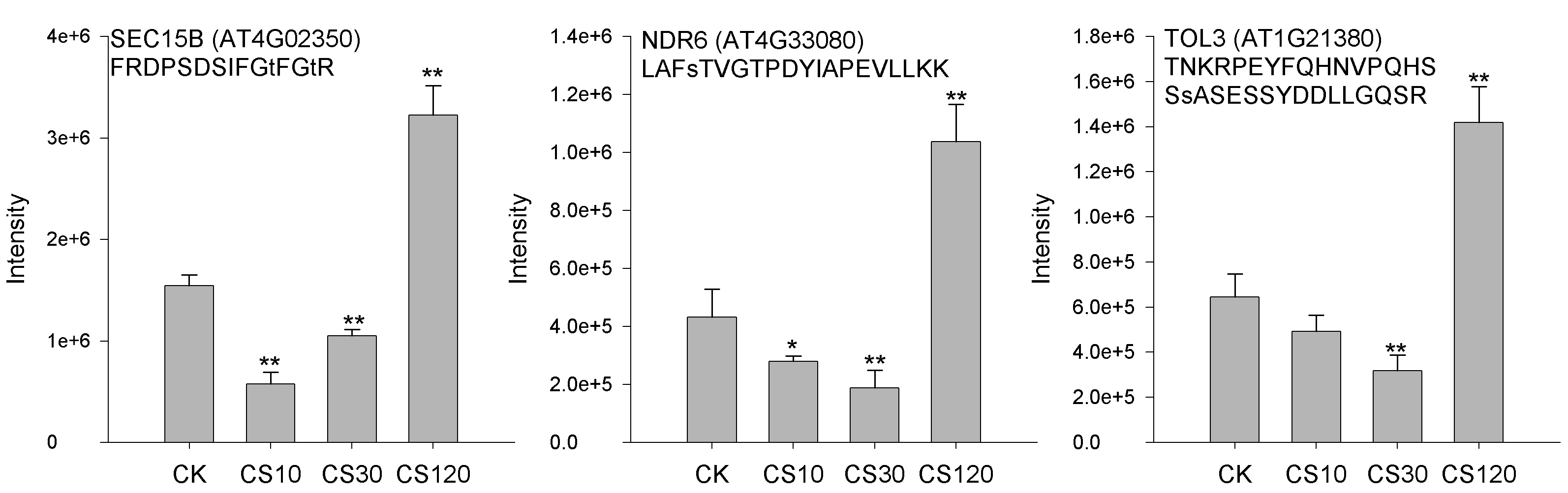

Supplement: Supplementary file 1 [file ijms-22-12856-s001.zip › Figure S2. Time-course profiling of three phosphopeptides which were initially down-regulated and subsequently up-regulated upon cold stress..png]

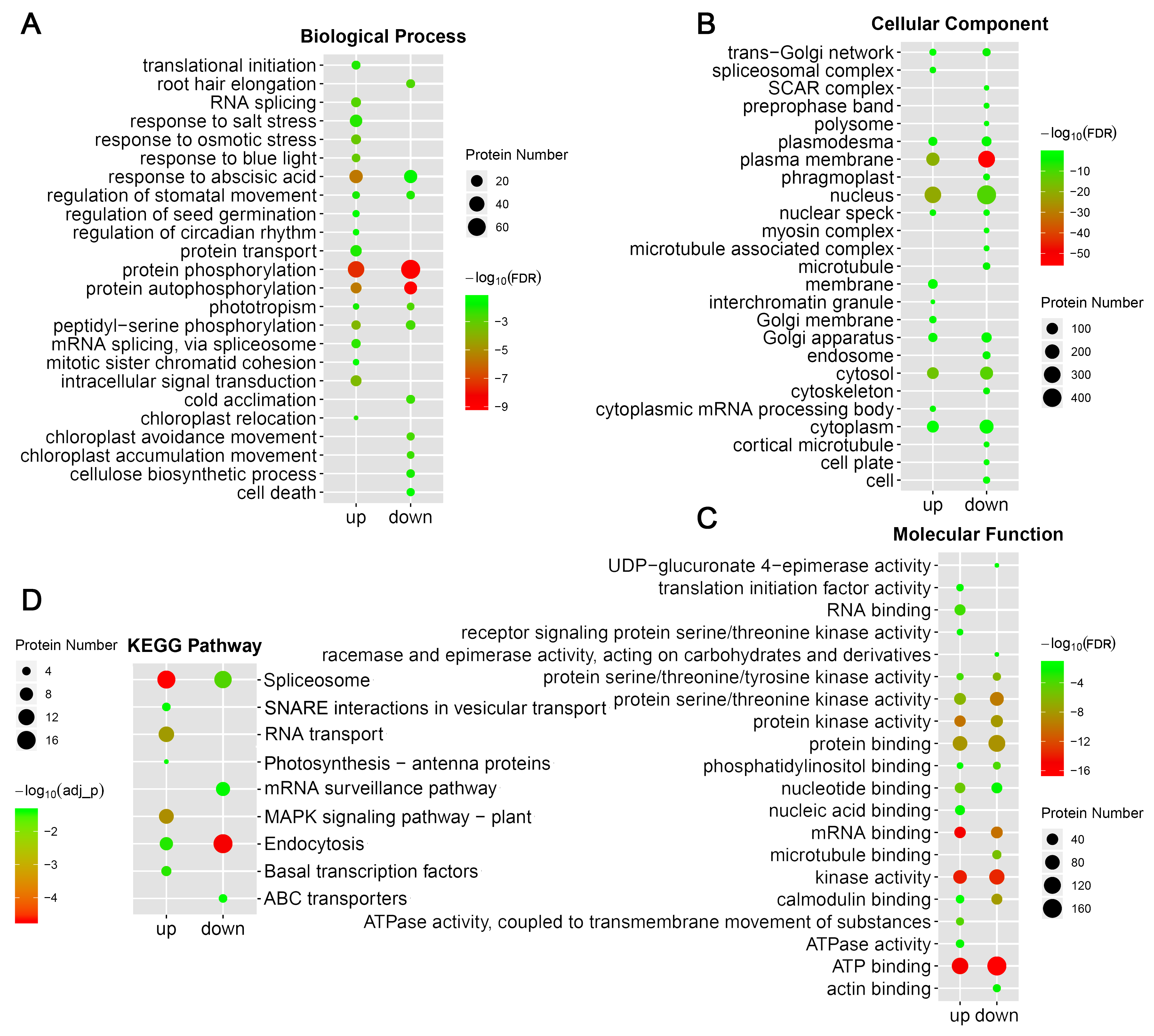

Supplement: Supplementary file 1 [file ijms-22-12856-s001.zip › Figure S3. GO enrichment (A, B, C) and KEGG pathway (D) analyses of proteins containing up-accumulated and down-accumulated phosphopeptides in response to cold stress..png]

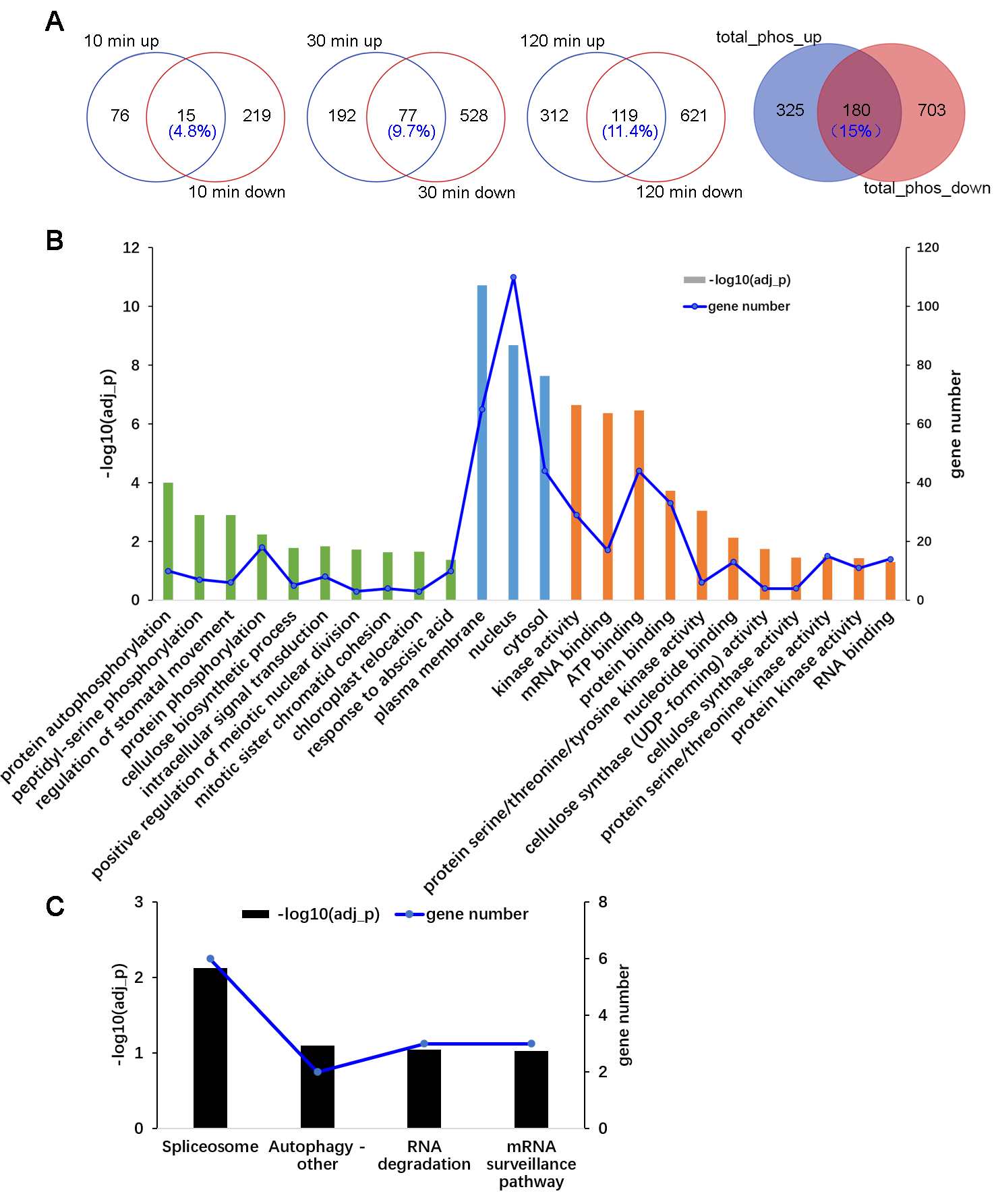

Supplement: Supplementary file 1 [file ijms-22-12856-s001.zip › Figure S4. Phosphoproteins contain both up-regulated and down-regulated phosphopeptides upon cold stress.png]

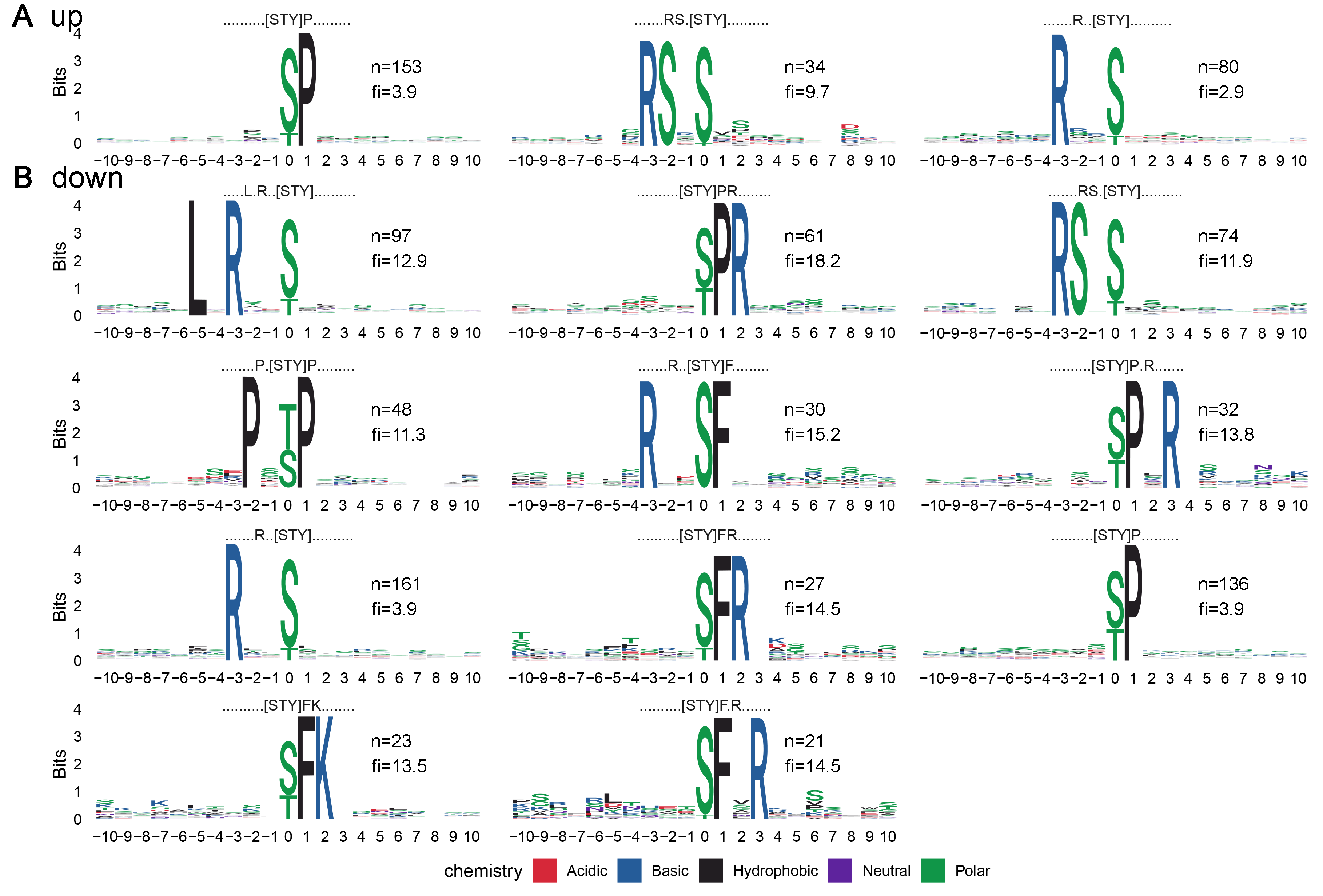

Supplement: Supplementary file 1 [file ijms-22-12856-s001.zip › Figure S5. Phosphorylation motif analysis of up-regulated (A) and down-regulated (B) phosphopeptides upon cold stress.png]

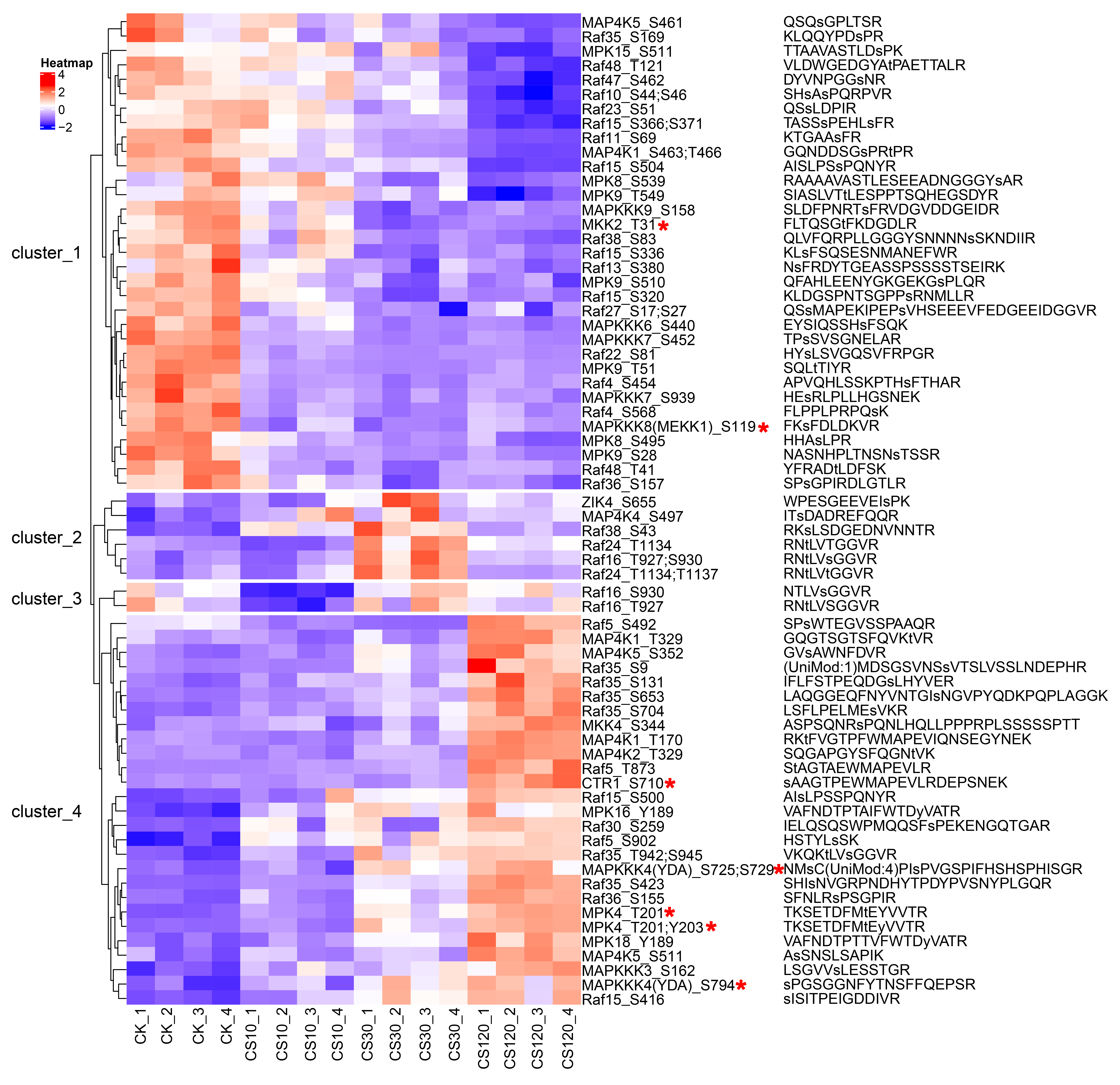

Supplement: Supplementary file 1 [file ijms-22-12856-s001.zip › Figure S9. Heatmap diagram showing the cold-responsive phosphopeptides involved in MAPK cascades.png]
